# Exploring Agro-Industrial By-Products: Phenolic Content, Antioxidant Capacity, and Phytochemical Profiling via FI-ESI-FTICR-MS Untargeted Analysis

**DOI:** 10.3390/antiox13080925

**Published:** 2024-07-30

**Authors:** Itzel Yoali Hernández-Montesinos, David Fernando Carreón-Delgado, Oxana Lazo-Zamalloa, Lilia Tapia-López, Minerva Rosas-Morales, Carlos Enrique Ochoa-Velasco, Paola Hernández-Carranza, Yair Cruz-Narváez, Carolina Ramírez-López

**Affiliations:** 1 Instituto Politécnico Nacional, Centro de Investigación en Biotecnología Aplicada, Carretera Estatal Santa Inés Tecuexcomac-Tepetitla, km 1.5, Tepetitla de Lardizábal, Tlaxcala 90700, Mexico; 2 Benemérita Universidad Autónoma de Puebla, Facultad de Ciencias Químicas, 4 Sur 104, Centro Histórico, Puebla 72000, Mexico; 3 Instituto Politécnico Nacional, Escuela Superior de Ingeniería Química e Industrias Extractivas, Av. Instituto Politécnico Nacional, Lindavista, Gustavo A. Madero, Ciudad de México 07700, Mexico

**Keywords:** agro-industrial by-products, phytochemical profile, FTICR-MS, phenolic compounds, antioxidant properties

## Abstract

This study investigates agro-industrial by-products as sources of bioactive compounds, particularly focusing on phenolic compounds known for their antioxidant properties. With growing interest in natural alternatives to synthetic antioxidants due to safety concerns, this study highlights the health benefits of plant-derived phenolic compounds in food preservation and healthcare products. Traditional and advanced analytical techniques were used to obtain phytochemical profiles of various residue extracts, including espresso (SCG) and cold-brew spent coffee grounds (CBCG), pineapple peel (PP), beetroot pomace (BP), apple pomace (AP), black carrot pomace (BCP), and garlic peel (GP). Assessments of total phenolic content (TPC), total flavonoid content (TFC), and antioxidant capacity (AC) supported their revalorization. CBCG showed the highest TPC, TFC, and AC. TPC content in by-products decreased in the order CBCG > SCG > GP > BCP > PP > AP > BP, with a similar trend for TFC and AC. Phytochemical profiling via FI-ESI-FTICR-MS enabled the preliminary putative identification of a range of compounds, with polyphenols and terpenes being the most abundant. Univariate and multivariate analyses revealed key patterns among samples. Strong positive correlations (Pearson’s R > 0.8) indicated significant contribution of polyphenols to antioxidant capacities. These findings highlight the potential of agro-industrial residues as natural antioxidants, advocating for their sustainable utilization.

## 1. Introduction

The concern regarding the use of synthetic antioxidants such as butylated hydroxytoluene (BHT) and butylated hydroxyanisole (BHA) has prompted the exploration of safer alternatives from natural sources [1]. Phenolic compounds, abundant in plant extracts, have been extensively studied for their antioxidant and antimicrobial properties, as well as health benefits. However, their widespread application faces challenges related to their availability and the adequacy of starting materials for industrial extraction [2]. To address these challenges, researchers have turned their attention to abundant but often overlooked agricultural by-products. Repurposing these by-products for bioactive compounds extraction could help mitigate environmental issues while contributing to sustainable agriculture and improved food systems [3].

Coffee, a globally consumed beverage, holds significant economic importance but also contributes to a substantial waste stream. Spent coffee grounds have been found rich in bioactive compounds whose presence and concentration are influenced by the brewing method [4]. Industrial pineapple processing also produces significant amounts of residues, presenting an opportunity to utilize by-products like peels to extract bioactive compounds [5]. Apple pomace, a residual from juice extraction, is esteemed for its phenolic richness and health benefits [6], while beetroot pomace garners attention for its phenolic content and antioxidant properties [7]. Additionally, carrots, with their diverse colors reflecting unique chemical compositions, offer various attributes, with black carrots notably boasting superior antioxidant activity and anthocyanin content [8]. Black carrot concentrate generates a pomace waste stream [9]. Finally, despite often being overlooked, garlic peel extracts are rich in phenolic compounds, displaying promising antioxidant and antimicrobial activities [10,11]. This study focuses on evaluating the phenolic content and antioxidant activities of ethanolic extracts derived from these agro-industrial residues.

In addition to traditional assays for phenolic content and antioxidant activity, this study employs direct injection ion cyclotron Fourier transform mass spectrometry (FT-ICR-MS) untargeted analyses to profile the metabolites present in the extracts. FT-ICR-MS offers ultra-high resolution and ultra-high mass accuracy, making it a powerful tool for metabolite profiling in complex mixtures [3]. This study aims to serve as an exploratory analysis to provide insights into the bioactive compounds of these by-products to promote their revalorization.

## 2. Materials and Methods

### 2.1. Agro-Industrial By-Product Materials, Reagents, and Solvents

All agro-industrial samples were provided by companies or local agribusiness from the states of Puebla and Tlaxcala, Mexico. Spent coffee grounds (SCG) and cold-brew coffee grounds (CBCG) samples were collected from the same coffee shop, resulting from espresso (92–95 °C, 15–25 s) and cold-brew extraction (full immersion method, 4 °C, 7 h) of medium roast arabica coffee, respectively. Pineapple peel (PP) samples were acquired from a pineapple processing plant that produces cut-fresh and dehydrated fruit. Beetroot pomace (BP) and apple pomace (AP) samples were obtained from a local juice shop. Black carrot pomace (BCP) samples were produced from juice extraction using a domestic juice extractor. Garlic peel (GP) samples were obtained as a by-product of a garlic paste producing plant and were composed of a mixture of external and internal garlic bulb peels. All samples were air-dried at 40 °C for 18 h, ground, sieved (2 mm mesh), and stored in dark glass containers at 4 °C until further utilization. All chemical reagents and solvents used in this study were purchased from Sigma-Aldrich, Inc. (Toluca, Mexico) and J.T. Baker (Mexico City, Mexico), respectively. 

### 2.2. Extract Preparation

Agro-industrial by-product samples were macerated with 75% ethanol at 53 °C for 77 min using a sample/solvent ratio of 1:25 (*w*/*v*), based on previous studies [11]. After extraction time concluded, extracts were filtered using Whatman N°4 paper and evaporated to dryness using a rotary evaporator. Dry extracts were stored in dark glass containers at 4 °C.

### 2.3. Total Phenolic Content (TPC) and Total Flavonoid Content (TFC) Determinations

Absorbances were measured using a Multiskan GO microplate spectrophotometer (Thermo Fisher Scientific, Waltham, MA, USA). To ensure dilution and stability of both polar and non-polar compounds, dry extracts were previously diluted in 2% (*v*/*v*) dimethyl sulfoxide (DMSO). Final concentration was adjusted to 1 mg/mL and results were expressed as milligrams of standard equivalents per gram of dry extract based on calibration curves (Supplementary Table S1).

For TPC, 100 µL of each extract sample were mixed with 100 µL of Folin–Ciocalteu reagent (0.1 N) and 1.5 mL of distilled water. The mixture was allowed to stand for 10 min, followed by the addition of 300 µL of 20% (*w*/*v*) sodium carbonate (Na_2_CO_3_). The resulting mixture was then incubated for 1 h at room temperature (22 ± 3 °C) in the dark. Absorbance was measured at 765 nm and results were expressed as milligrams of gallic acid equivalents per gram of dry extract (mg GAE/g) based on the gallic acid calibration curve (at a linearity range of 0–0.35 mg/mL, with the equation y = 2.795x + 0.015).

For TFC, 200 µL of each extract sample was mixed with 400 µL of distilled water and 60 µL of 5% (*w*/*v*) NaNO_2_ and incubated for 5 min. Subsequently, 60 µL of 10% AlCl_3_ (*w*/*v*) was added. After 6 min, 400 µL of NaOH 1 N and 400 µL of distilled water were added. The mixture was then allowed to rest for 5 min in darkness before measuring absorbance at 510 nm. Results were expressed as milligrams of quercetin equivalents per gram of dry extract (mg QE/g) based on the quercetin calibration curve (at a linearity range of 0–0.7 mg/mL, with the equation y = 1.49x − 0.022).

### 2.4. Antioxidant Assays

The antioxidant capacities of the agro-industrial by-product extracts were assessed using 1,1-diphenyl-2-picrylhydrazyl (DPPH) [11], 2,20-azino-bis(3-ethylbenzothiazoline-6-sulfonate) (ABTS) [12], and ferric reducing antioxidant power (FRAP) assays [13]. Dry extracts were diluted with adequate volumes of 2% (*v*/*v*) DMSO to produce extracts with varying concentrations up to 6 mg/mL. 

For the DPPH assay, various concentrations of the extract (50 µL) were combined with DPPH ethanolic solution (150 µL, 90 µM) and incubated at 37 °C in the absence of light for 30 min. The absorbance was read at 517 nm. In the ABTS assay, a stock solution of ABTS radical was prepared by mixing ABTS (7 mM) and potassium persulfate (2.45 mM) in equal volumes and allowing the mixture to incubate in darkness for 12 h. This stock solution was diluted with distilled water to achieve an absorbance of 0.70 ± 0.02 at 734 nm. Subsequently, different concentrations of each extract (10 µL) were mixed with diluted ABTS (190 µL) and incubated in darkness for 7 min. The absorbance was measured at 734 nm. The radical scavenging percentage was determined by assessing the change in absorbance resulting from the reduction in DPPH and ABTS compared with the control. IC_50_ values, representing the concentration of extracts necessary to inhibit 50% of radicals, were determined by plotting extract concentrations against radical scavenging percentages. FRAP assay was employed to assess the antioxidant capacity of samples by measuring their ability to reduce ferric iron (Fe^3+^) to ferrous iron (Fe^2+^). Fresh FRAP reagent was added to each well in a microplate along with 10 µL of each extract at 1 mg/mL. The absorbance was measured after incubation at 37 °C for 30 min. For Trolox-equivalent antioxidant capacity (TEAC) determination, all assays were also conducted using 6-hydroxy-2,5,7,8-tetramethylchroman-2-carboxylic acid (Trolox) as a standard to express the results as µmol of Trolox equivalents per gram of dry extract (µmol TE/g).

### 2.5. Untargeted Phytochemical Profiling by FI-ESI-FTICR-MS 

Untargeted phytochemical profiling analyses were conducted for each agro-industrial by-product extract using a Bruker Solarix XR 7T (Bruker Daltonics, Bremen, Germany) Fourier transform ion cyclotron resonance mass spectrometer (ICR-MS). The instrument was equipped with a direct infusion electrospray ionization (ESI) source operating via flow injection (FI) and calibrated with sodium trifluoroacetate standard. Extract samples were prepared for FI-ESI-FTICR-MS analyses by diluting 1 mg of dry extract in 1 mL of water/ethanol/acetic acid (24:74:2 *v*/*v*/*v*) and filtering through a 0.22 µm Merck syringe filter. Extract samples were injected in triplicate via a Hamilton 250 μL syringe at a flow rate of 120 μL/h, employing both positive and negative ESI modes under the following parameters: 3500 V, 2499 nA capillary; −500 V, 62.207 nA end plate offset, and 8 M resolving power. Full-scan MS data were acquired across an *m*/*z* range of 43–3000, averaging 100 scans, with an accumulation time of 0.1 s. The source gas tune comprised N_2_, nebulized at 2 bars, with a dry gas flow of 4 L/min and a temperature of 180 °C. Data processing was performed by Compass DataAnalysis v.6.0 software (Bruker Daltonics), and MetaboScape 2022 b v.9.0.1 (Bruker Daltonics) was employed for the putative annotation of metabolites. Theoretical masses were calculated using ChemCalc [14].

### 2.6. Statistical Analyses

All experiments were conducted in triplicate. Values were expressed as mean ± standard deviation. One-way statistical analyses of variance (ANOVAs), with mean differences assessed through Tukey’s post hoc test (α = 0.05), were carried out using Minitab 19 for Windows (Minitab Inc., State College, PA, USA). Plots, Pearson’s correlations, and IC_50_ calculations were performed using GraphPad Prism 9 for Windows (GraphPad Software, San Diego, CA, USA), with data fitted to a three-parameter logistic dose–response regression model. Univariate analysis in phytochemical profiling, principal component analysis (PCA), hierarchical clustering, and partial least squares discriminant analysis (PLS-DA) were conducted using the web tool MetaboAnalyst 6.0 (https://www.metaboanalyst.ca) (accessed on 11 april 2024), using sum normalized and Pareto scaled FI-ESI-FTICR-MS data (peak intensities with mass error up to 20 ppm in each ESI mode). PLS-DA models’ validation tests are shown in Supplementary Figure S1.

## 3. Results and Discussion

### 3.1. Total Phenolic Content, Total Flavonoid Content, and Antioxidant Capacity 

**Figure 1:**
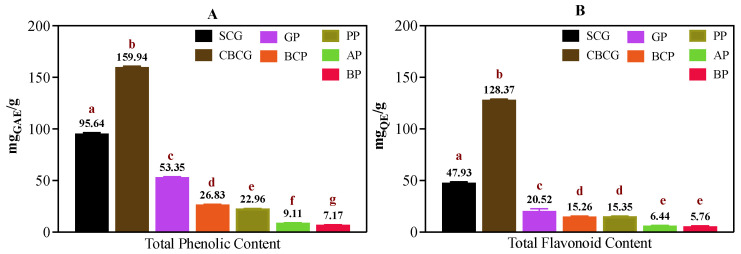
(**A**) Total phenolic content and (**B**) total flavonoid content of agro-industrial by-products extracts. SCG—spent coffee grounds, CBCG—cold-brew spent coffee grounds, GP—garlic peel, BCP—black carrot pomace, PP—pineapple peel, AP—apple pomace, BP—beetroot pomace. Different lower-case letters indicate significant differences between extracts (Tukey’s post hoc test, α = 0.05).

**Figure 2:**
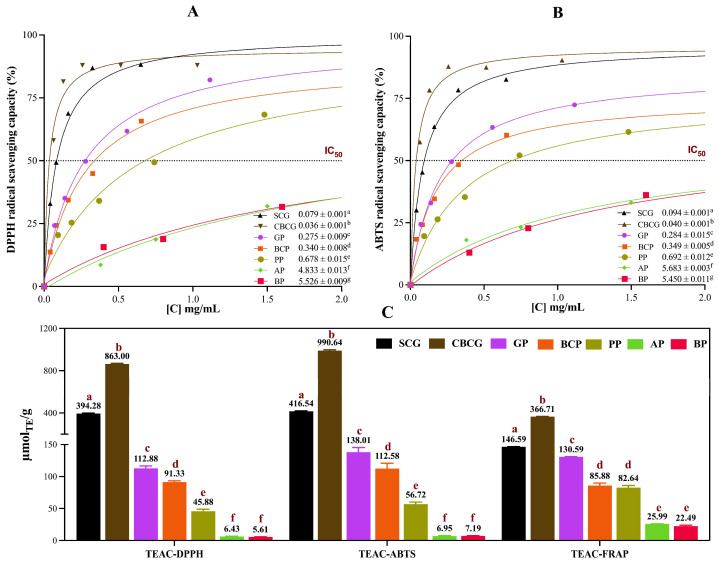
Antioxidant capacity of agro-industrial by-products extracts. (**A**) DPPH and (**B**) ABTS dose–response curves following three-parameter regression models for IC_50_ radical scavenging capacity determination. (**C**) Trolox equivalent antioxidant capacity (TEAC) values. SCG—spent coffee grounds, CBCG—cold-brew spent coffee grounds, GP—garlic peel, BCP—black carrot pomace, PP—pineapple peel, AP—apple pomace, BP—beetroot pomace. Different lower-case letters indicate significant differences between extracts (Tukey’s post hoc test, α = 0.05).

Assessments of TPC, TFC, and AC were conducted for each of the studied agro-industrial by-product extracts, as depicted in Supplementary Table S2. Figure 1 illustrates the average TPC (A) and TFC (B) values observed in each set of samples, while Figure 2C presents the corresponding AC data obtained from DPPH, ABTS, and FRAP assays. Additionally, Figure 2A,B depict extract concentration vs. DPPH and ABTS radical scavenging percentage curves, respectively, from which IC_50_ values were calculated.

It can be observed in Figure 1A that the cold-brew coffee grounds (CBCG) and spent coffee grounds (SCG) extracts were the ones with the highest TPC; the levels were in the range of 159.94 to 95.64 mg GAE/g, respectively. These extracts also resulted in the highest TFC, exhibiting levels of 128.37 and 47.93 mg QE/g (Figure 1B). There were no significant differences (*p* > 0.05) observed in TFC levels between the black carrot pomace (BCP) and pineapple peel (PP) samples, nor between the apple pomace (AP) and beetroot pomace (BP) samples, although the TPC levels differed significantly (*p* < 0.05) among all samples in the following decreasing order: CBCG > SCG > GP > BCP > PP > AP > BP. For TFC, levels followed the same tendency as TPC for all samples.

AC of extracts (Figure 2C) followed overall a very similar tendency as TPC (Figure 1A), with the extracts from coffee by-products being the ones with the highest AC and decreasing in the order of CBCG > SCG > GP > BCP > PP > AP > BP. Regarding the samples with the highest TPC, TFC, and AC levels, it is noteworthy that both CBCG and SCG samples were by-products coming from the same starting material, which was medium roast Arabica coffee, the brewing method being the differentiating point between them. SCG were obtained after espresso extraction, which involved high water temperatures and short extraction times. Conversely, CBCG were obtained through a cold-brew method characterized by prolonged extraction times and low temperatures. A recent analysis of spent coffee grounds from various initial brewing processes reported higher TPC levels in spent grounds from cold brew compared with espresso, the reported levels being 420.5 and 313.5 mg GAE/L, respectively. Additionally, higher AC values, as measured by DPPH, ABTS, and FRAP assays, were observed in cold-brew spent grounds compared with espresso spent grounds, consistent with our findings [4].

The above findings can be attributed to the fact that the extraction of bioactive compounds assessed on these samples represents a secondary extraction of the compounds remaining after the brewing of coffee beverages. Consequently, a fraction of the bioactive molecules initially present in the coffee grounds end up in the beverages or are lost through volatilization [15]. Therefore, it is expected to observe higher levels of bioactive compounds in samples subjected to a less efficient primary extraction process. Studies have evidenced that the TPC in espresso is generally higher compared with cold brew (CB), resulting in a larger fraction of unextracted compounds remaining in the CB spent grounds [16,17]. Another study corroborated these findings, demonstrating that classical espresso brewing methods achieve higher concentrations of bioactive compounds per milliliter than CB [18]. It has been suggested that hot brew (HB) coffee, especially at increased roasting levels, tends to have a higher TPC than its CB counterpart. This is attributed to the enhanced solubility of antioxidant compounds like melanoidins in hot water, which benefits from temperature-induced solubility enhancements [19]. 

As depicted in Figure 1 and Figure 2, after the spent coffee grounds samples, garlic peel (GP) exhibited the highest TPC (53.35 mg GAE/g), TFC (20.52 mg QE/g), and AC levels (for µmol TE/g, the results were 112.88 for DPPH, 138.01 for ABTS, and 130.59 for FRAP). These results are in agreeance with previously reported data on GP extracts obtained with 70% ethanol at 50 °C for 1 h, consisting of levels of TPC of 50.41 mg GAE/g, TFC of 47.58 mg QE/g, DPPH-TEAC of 82.59 µmol TE/g, and ABTS-TEAC of 199.07 µmol TE/g [11]. The antioxidant activity of garlic peel is mostly attributed to its high phenolic content, which helps scavenge free radicals and prevent oxidative damage. Black carrot is recognized as a rich source of polyphenols with high bioaccessibility levels [8]. However, it has been evidenced that processing can lead to a reduction in both TPC and antioxidant capacity [9]. Despite this, black carrot by-products such as peel and pomace remain substantial sources of polyphenols. Slightly lower than our results (Figure 1) but consistent in magnitude, TPC values of 2.30 mg GAE/g have been reported for black carrot pomace [20]. Under optimized conditions using microwave-assisted extraction (MAE), the TPC of a black carrot pomace was found to be 264.9 mg GAE/100 mL, while values of AC (FRAP) ranging from 2.82 to 14.24 µmol TE/mL were reported [21]. 

For PP extracts, various studies have reported different TPC values. For instance, values ranging from 219.75 to 405.06 mg GAE/ 100 g were found in ultrasound-assisted extractions [22]. TPC of 208.899 mg GAE/kg was reported for a methanolic extract [23]. These differences demonstrate the influence of extraction methods, solvents, and operational conditions on the phenolic compound yield from plant material. In the case of AP extracts, a study reported the highest TPC levels in aqueous extracts using a water-to-pomace ratio of 20:1 at 90 °C for 15 min, yielding 1148 µgGAE/g [24]. This value was slightly lower but comparable to the TPC level found in our study (Figure 1). Moreover, a TPC of 9.95 mg GAE/g was reported for an extract prepared using a 50% water/ethanol solution at 60 °C [25]. Remarkably similar to our findings, this result may be attributed to the similar extraction conditions employed. Regarding AC, FRAP activity of 5.07 mmol ascorbic acid equivalents (AAE)/100 g and DPPH scavenging activity of 3.74 mmol Trolox/100 g were reported [25]. 

BP is another rich source of phenolics. TPC of 376.4 mg GAE/g of dry extract has been reported in a study analyzing ethanolic BP extracts [26]. The authors determined AC by DPPH assay and concluded that the reducing power of the beetroot pomace extracts increased with increasing concentrations. They reported DPPH scavenging activities as IC_50_ ranging from 0.133 mg/mL to 0.275 mg/mL. Agreeing with our findings, significant correlation was observed between phytochemical components and scavenging activity. Recognizing that the employed extraction method may not be optimal for all samples, potentially affecting the results, in-depth studies are needed to compare various extraction techniques to optimize the recovery of specific compounds from each by-product. 

### 3.2. Untargeted Phytochemical Profiling by FI-ESI-FTICR-MS 

An untargeted metabolomic strategy was employed to comprehensively screen and profile the phytochemical compounds extracted from the agro-industrial by-product samples through FI-ESI-FTICR-MS analysis. It is important to note that direct injection in high-resolution mass spectrometry lacks the retention time and fragmentation pattern information that is necessary for confidently identifying molecular structures. However, the ultra-high resolution of FT-ICR-MS provides accurate mass data and allows for specific formula assignment down to sub-part-per-billion (ppb) levels. This enables the preliminary putative characterization of isotopic metabolites. Our data were deemed adequate for the putative characterization of compounds (level 3), particularly in identifying compound classes based on characteristic physicochemical properties or spectral similarity to known compounds within the same class [27].

**Figure 3:**
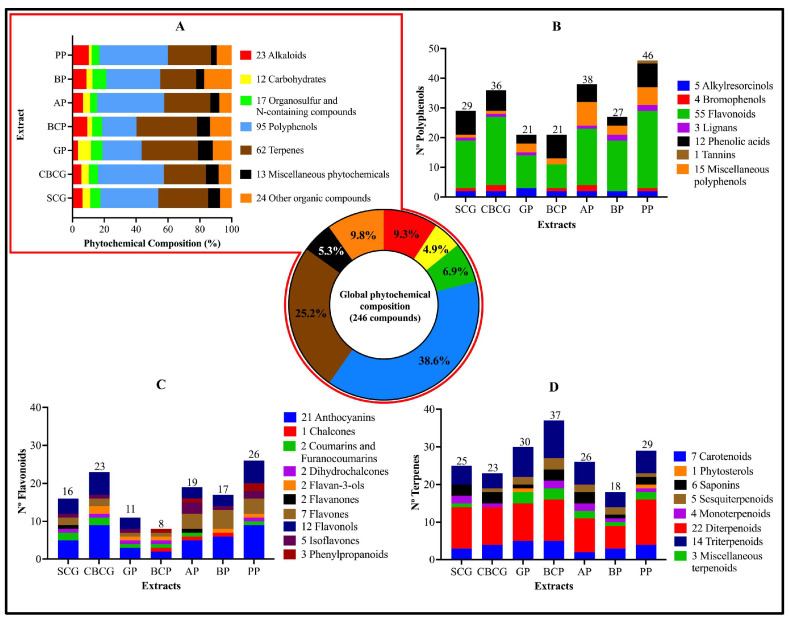
Phytochemical profile charts. (**A**) Percentage composition of each compound family in each agro-industrial by-product extract (bar plot) and global composition (pie chart), (**B**) number of polyphenols, (**C**) number of flavonoids, and (**D**) number of terpenes and their sub-classes found in each extract. SCG—spent coffee grounds, CBCG—cold-brew spent coffee grounds, GP—garlic peel, BCP—black carrot pomace, PP—pineapple peel, AP—apple pomace, BP—beetroot pomace.

A total of 4729 and 6151 ions (peaks) were identified in both positive and negative electrospray ionization modes (ESI+, ESI−), and 734 compounds were annotated from the databases (284 in ESI+ mode and 450 in ESI− mode). Matches with a mass error under 20 ppm were selected for untargeted metabolomic analysis, resulting in 99 from ESI+ and 160 from ESI− (Table 1). Identified peaks with mass error up to 20 ppm in both ESI modes are shown in Supplementary Tables S3 and S4. This assessment revealed the presence of a diverse array of phytochemicals, which were classified into the following families: alkaloids, carbohydrates, polyphenols, terpenes, organosulfur and N-containing compounds (such as glucosinolates, isothiocyanates, amino acids, proteins, nucleosides, and nucleotides), and miscellaneous phytochemicals and other organic compounds (including fatty acids, lipids, organic acids, and vitamins). Considering that 13 phytochemicals were identified in both ionization modes (5 polyphenols, 5 terpenes, 2 lipids, and 1 carbohydrate), 246 different phytochemicals with a mass error under 20 ppm were considered for the phytochemical profiling analysis presented in Figure 3. 

As can be seen in Figure 3A (pie chart), the global composition of all extracts consisted of 23 alkaloids, 12 carbohydrates, 17 organosulfur and N-containing compounds, 95 polyphenols, 62 terpenes, 13 miscellaneous phytochemicals, and 24 other organic compounds. Polyphenols and terpenes were the most abundant families of the matched phytochemicals, comprising 38.6 and 25.2% of the global composition, respectively. Polyphenolic metabolites were also classified into the following classes: alkylresorcinols, bromophenols, flavonoids, lignans, miscellaneous polyphenols, phenolic acids, and tannins (flavonoids being the major phenolic class in all extracts) (Figure 3B). Consequently, the annotated flavonoids were classified into the sub-classes of anthocyanins, chalcones, dihydrochalcones, flavan-3-ols, flavanones, flavones, flavanols, isoflavones, phenylpropanoids, coumarins, and furanocoumarins (Figure 3C), where anthocyanins predominated among the flavonoid classes. Accordingly, compounds of the terpene family were classified into classes as carotenoids, phytosterols, saponins, sesquiterpenoids, monoterpenoids, diterpenoids, triterpenoids, and miscellaneous terpenoids (Figure 3D), with a higher presence of terpenoids. Regarding the composition of each extract, it was observed that the profiles of the coffee samples (SCG and CBSG) were similar, with polyphenols constituting over 30 percent of the composition in both extracts, followed by terpenes. 

Also, GP and BCP extracts displayed comparable phytochemical compositions, with a greater abundance of terpenes over polyphenols and the other compounds. In contrast, AP, BP, and PP extracts showcased a predominance of polyphenols over terpenes. To explore the phenolic and terpene composition within the sub-products, an analysis of the top abundant phenolic and terpene peaks for each extract was conducted, as detailed in Table 2 and Table 3. 

Ferulic acid is a hydroxycinnamic acid derivative and is one of the most abundant phenolic compounds found in plants [28]. A significant number of ferulic acid ester derivatives, such as campesteryl ferulate and methylcholestanol ferulate, were identified in the studied samples. These compounds are esters formed by the conjugation of ferulic acid with sterols. Ferulic acid derivatives are commonly found in grains but their presence has also been documented in other plant tissues, including roots [29]. Furthermore, ferulic acid and its derivatives possess a wide range of biological activities, including anticancer, antibacterial, anticarcinogenic, and anti-inflammatory activity [28].

Polyphenols exhibiting higher abundances in SCG extract included kaempferol3-o-(6-malonyl-glucoside), nonadecatrienyl-resorcinol, methylcholestanol ferulate, cardanol, and delphinidin-3-arabinoside. Similarly, in CBCG extract, the polyphenols with higher abundance comprised kaempferol3-O-(6-malonyl-glucoside), nonadecatrienyl-resorcinol, 6″-O-acetylglycitin, paradisin B, and delphinidin-3-arabinoside. Kaempferol, a flavonoid known for its antioxidant properties, and its glycosidic derivative, kaempferol-3-O-glucoside, have been identified in coffee [30]. Isoflavonoid 6″-O-acetylglycitin was detected in Geisha coffee beans [31]. GP extract contained bergapten, nonadecatrienyl-resorcinol, kaempferol-3-O-galactoside7-O-rhamnoside, and epicatechin-(2α-7)(4α-8)-epicatechin 3-O-galactoside as its prominent polyphenols. Bergapten has been identified in the peels of Sulmona red garlic [32] and the presence of kaempferol in garlic has been reported [33]. Epicatechin has been found in extracts of plants of the *Allium* genus such as onions [34]. BCP extract showcased campesteryl ferulate, methylcholestanol ferulate, nonadecatrienyl-resorcinol, nobiletin, and bergapten among its polyphenolic compounds with higher abundance. In a study on the furocoumarin contents in popularly consumed foods in the U.S., the presence of bergapten in carrots was addressed [35]. Storing root vegetables below 4 °C prevented an increase in furanocoumarin levels including bergapten, which may occur when the crop is kept at higher temperatures [36]. AP extract displayed benzoic acid, echinatin, 7-hydroxymatairesinol, tetramethylscutellarein, and bergapten as its dominant polyphenols. Benzoic acid has been found to be naturally occurring as an antifungal compound associated with the resistance of apples to rotting [37] and is utilized as a preservative in apple juice [38]. Furthermore, BP extract was characterized by kaempferol 3-O-(6-malonyl-glucoside), epicatechin-(2α-7)(4α-8)-epicatechin 3-O-galactoside, nonadecatrienyl-resorcinol, cyanidin 3-(feruloyl)(sinapoyl)-triglucoside-5-glucoside, and methylcholestanol ferulate as its polyphenols with major abundance.

Epicatechin has been addressed as a main flavonoid present in beetroot extracts [39,40,41], possessing several desirable biological activities, including antioxidant, anti-inflammatory, hepatoprotective, and antitumor properties [40]. An HPLC analysis of BP extract showed that catechin was the most abundant flavonoid, followed by myricetin and epicatechin, with small quantities of apigenin, luteolin, quercetin, and isorhamnetin also detected [42]. LC-MS analysis of beetroot peel extract also identified myricetin, epicatechin, epigallocatechin, catechin, umbelliferone, luteolin, rutin, hesperetin, and kaempferol [43]. Accordingly, kaempferol and epicatechin derivatives were found to be abundant in BP extract. Lastly, PP extract exhibited kaempferol3-o-(6-malonyl-glucoside), nonadecatrienyl-resorcinol, bergapten, and methylcholestanol ferulate as noteworthy polyphenols. Kaempferol has been previously identified in pineapple peels [44].

Regarding terpenes, sterols such as cycloartenol, 24-methylenecyloartanol, cycloeucalenol, obtusifoliol, citrostadienol, 24-methylenelophenol, stigmasterol, sitosterol, campesterol, stigmastanol, and campestanol have been previously reported in Arabica coffee [45]. Coffee is also an important source of diterpenes, such as cafestol and kahweol, predominantly found in unfiltered brews such as espresso [46]. HPLC–DAD was used for the analysis of some diterpene esters in Arabica coffee brew where cafestol stearate was quantified [47]. In the present work, cafestol stearate and a ferulated derivative of stigmastanol were identified in SCG and CBCG extracts, as along with diterpene fatty acid esters, namely caffarolides B, F, and G, which have been isolated from green beans of Arabica coffee before [48]. Neoxanthin, a chloroplast pigment, has been also previously identified in coffee pulp and peel [49]. Terpenes are found in carrots mainly in the form of glycosides as precursors of volatile compounds [50]. BCP extract resulted in abundances of terpenoids and sesquiterpenoids such as lettucenin A. In a study on the identification and characterization of terpene synthases potentially involved in the formation of volatile terpenes in carrot, more than 41 terpenes were detected via SPME−GC−MS, among which 23 were identified as monoterpenes and 17 as sesquiterpenes [51]. Saponin terpenoids and betulinic acid were abundant in AP extract. Triterpenic acids are commonly found in a variety of plants, either in their free acid form or as aglycones within triterpenoid saponins [52]. Betulinic acid has been found in various parts of the apple, although notably the higher concentrations have been encountered in the peels [53]. It was reported to have anti-inflammatory, antiviral, antibacterial, antimalarial, and antioxidant properties [54]. In a recent work on beetroot, 40 saponins across various varieties were identified via UHPLC-HRMS, demonstrating a high content of triterpene saponins, including soyasaponin III [55], which was identified in the BP extract. 

Carotenoids, including neoxanthin and flavoxanthin, are pigments found in various fruits, flowers, and vegetables such as tomato, carrot, pineapple, papaya, sunflower, saffron, and green leaves. They contribute to the vibrant yellow, orange, and red colors observed in plants and are widely used commercially as natural colorants and nutritional supplement ingredients [56]. Carotenoids exhibit antioxidant properties, with some being precursors of vitamin A [57]. These compounds are synthesized within chloroplasts. Notably, a PP analysis via HPLC-DAD-APCI-MS revealed a distinctive profile containing chloroplast-specific pigments such as β-carotene, violaxanthin, neoxanthin, and lutein [58], aligning with the carotenoid composition observed in the PP extract. GP extract also showed abundant carotenoids and triterpenoids, which have been reported in garlic extract along with steroids, flavonoids, alkaloids, saponins, tannins, and glycosides [59].

### 3.3. Univariate and Multivariate Analyses of Phytochemical Profiles in Agro-Industrial Sub-Products

Univariate and multivariate analyses, such as one-way ANOVA, Tukey’s post hoc analysis, PCA, and PLS-DA, were used as exploratory tools to uncover relationships and patterns among the extracts, revealing key features that contributed to variance in phytochemical profiles. These methods provided a detailed examination of the mass spectrometry data, facilitating interpretation and understanding of the phytochemical composition in each agro-industrial sub-product. 

**Figure 4:**
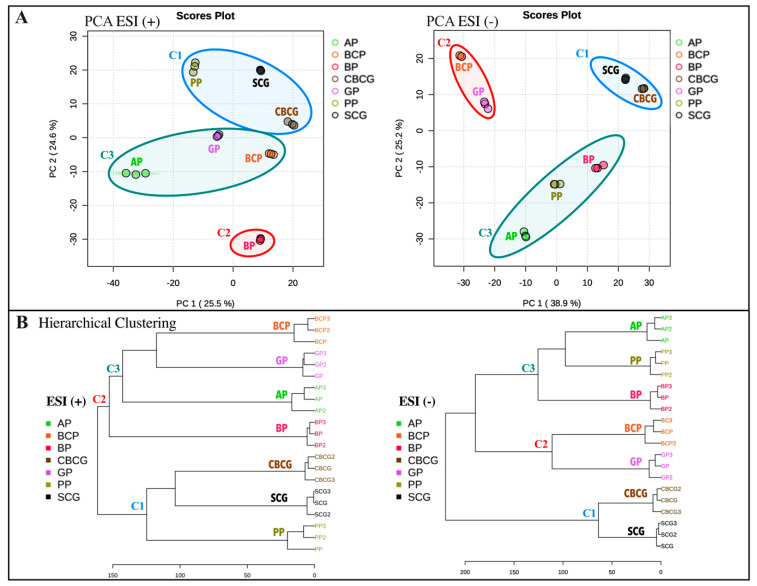
Overview of the primary untargeted metabolomic analysis from agro-industrial by-products extracts. (**A**) principal component analysis (PCA) score plots and (**B**) dendrograms from hierarchical cluster analysis (HiCA) using Euclidean distance and Ward linkage. FI-ESI-FTICR-MS positive (+) and negative (−) ESI modes. SCG—spent coffee grounds, CBCG—cold-brew spent coffee grounds, GP—garlic peel, BCP—black carrot pomace, PP—pineapple peel, AP—apple pomace, BP—beetroot pomace.

Figure 4 displays the PCA scores plots (A) alongside the hierarchical clustering dendrograms (B) of the ESI+ and ESI− datasets, offering an initial understanding of the data structure and clustering patterns. In both PCA score plots, hierarchical clustering of the samples is displayed. Replicates of each extract formed distinct clusters, evidencing the appropriateness of the selected analytical conditions. The resulting dendrograms revealed that, in ESI+ mode, the primary cluster initially grouped SCG, CBSG, and PP samples together, while subsequent clustering led to the separation of BP from the remaining samples. In the ESI− mode, the samples displayed improved distribution, with the main principal components (PC) explaining a greater variance (64.1%) compared with ESI+ (50.1%). The clusters were distinctly separated on the scatterplot, making them easier to distinguish. Similar to ESI+, the first cluster consisted of SCG and CBSG, distinct from a second cluster comprising GP and BCP. A third cluster included AP, PP, and BP, setting them apart from the first two clusters.

This analysis provides a structured method to group the extracts based on their similarities and differences, helping to categorize and interpret the variations observed among the different samples. As expected, coffee residues extracts (SCG, CBSG) clustered together since, as has been mentioned before, they come from the same source material, differing only in the brewing method. Interestingly, GP and BCP also exhibited similarities since they clustered together in both ionization modes, while the rest of extracts clustered differently depending on the ionization modes.

**Figure 5:**
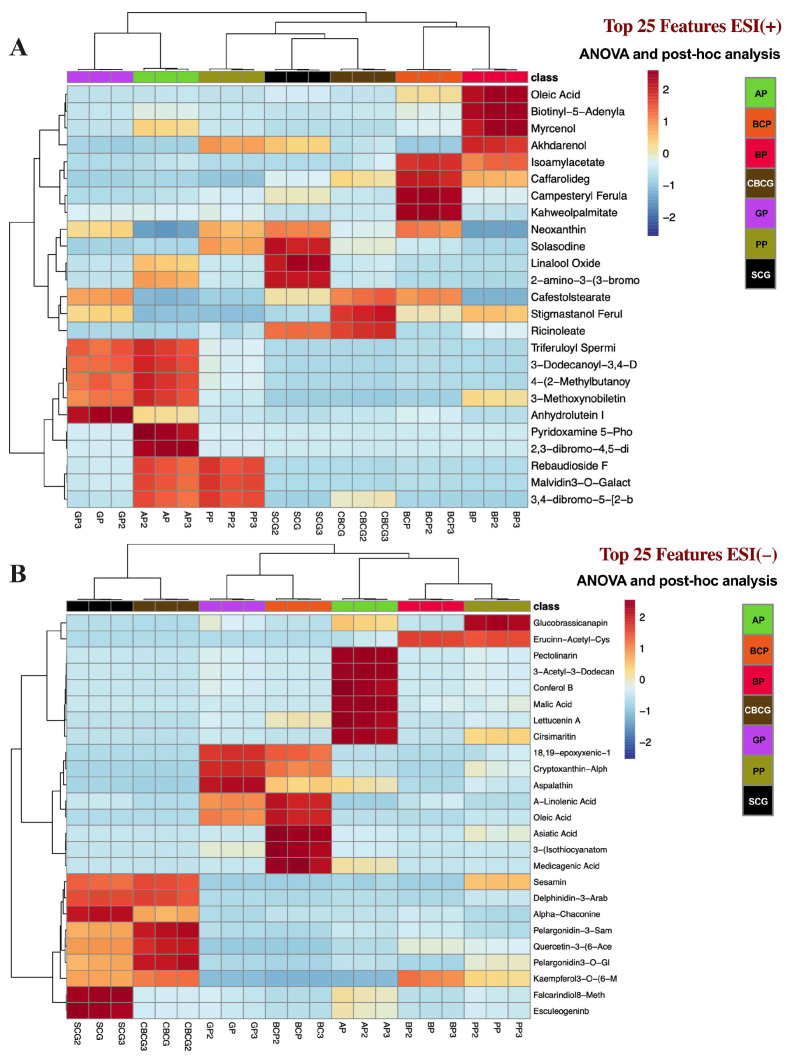
Heatmap analysis of the compounds detected in agro-industrial by-products extracts via by FI-ESI-FTICR-MS in (**A**) positive and (**B**) negative ESI modes that were significantly different in abundance (*p* < 0.05 after Tukey’s post hoc testing). Both features (rows) and samples (columns) were clustered and reordered by the similarity of the intensity patterns. Red zones indicate higher concentrations of compounds within samples. SCG—spent coffee grounds, CBCG—cold-brew spent coffee grounds, GP—garlic peel, BCP—black carrot pomace, PP—pineapple peel, AP—apple pomace, BP—beetroot pomace.

Additionally, heatmap analysis was conducted to identify the main differences in the phytochemical profiles of the extracts. Figure 5 displays color-coded 2D heatmaps generated through hierarchical clustering of features and samples using Euclidean distance and Ward’s method. These heatmaps showcase the top 25 features identified from one-way ANOVA and post hoc analysis in both ESI modes. This visualization offers insights into the distribution of the top phytochemicals among the extracts that were statistically different within each other (*p* < 0.05), highlighting compounds unique to specific samples and facilitating comparison of compound levels, with red zones indicating higher concentrations. 

**Figure 6:**
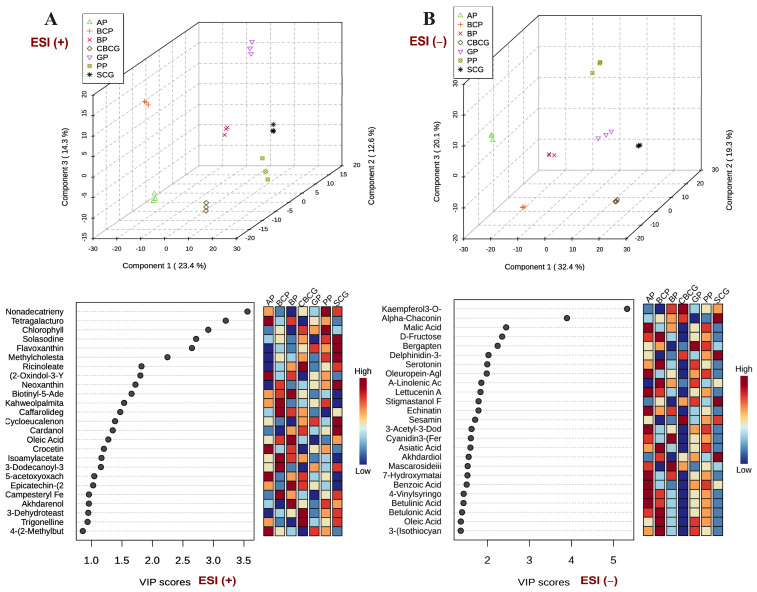
Partial least-squares discriminant analysis (PLS-DA) 3D score plots of differential metabolites detected in agro-industrial by-products extracts via FI-ESI-FTICR-MS in (**A**) positive and (**B**) negative ESI modes and their corresponding variable importance in projection (VIP) scores. SCG—spent coffee grounds, CBCG—cold-brew spent coffee grounds, GP—garlic peel, BCP—black carrot pomace, PP—pineapple peel, AP—apple pomace, BP—beetroot pomace.

Both 3D score plots in Figure 6 showed distinct clusters conformed by the replicates of each agro-industrial by-product extract. This clustering indicated high similarity within each extract group and significant differences between extracts based on their phytochemical compositions, validating the reproducibility and discriminative power of PLSDA in distinguishing between extracts, aiding in the identification of unique chemical signatures for each agro-industrial by-product. Features with VIP scores > 1 were considered especially influential in the separation achieved by PLSDA, suggesting their potential significance in defining the unique phytochemical profiles of each extract.

SCG samples exhibited higher levels of various polyphenols, including methylcholestanol ferulate and cardanol, as well as terpenes such as carotenoids (flavoxanthin, neoxanthin) and terpenoids (cycloeucalenone, stigmastanol ferulate, akhdardiol). Additionally, alkaloids like solasodine and α-chaconine were also more abundant in SCG samples. 

CBSG samples exhibited heightened levels of various polyphenols, notably kaempferol 3-O-(6-malonyl-glucoside) and sesamin, alongside an abundance of trigonelline (alkaloid) and lipidic metabolites such as ricinoleate and 3-dehydroteasterone. GP samples revealed a significant presence of a coumarin compound (bergapten). BCP samples resulted in an abundance of a phenolic acid (campesteryl ferulate) and terpenoids like caffarolide G and asiatic and betulonic acids. Additionally, an organosulfur compound (3-(isothiocyanatomethyl)-1H-indole), alongside other organic compounds such as fatty acids (A-linolenic, oleic) and carbohydrates (D-fructose, 3-dodecanoyl-3-isobutanoyl-4-(3-methylbutanoyl)-sucrose), and miscellaneous phytochemicals like kahweol palmitate and isoamyl acetate were notable in BCP samples.

PP samples demonstrated elevated levels of nonadecatrienyl-resorcinol (phenolic alkylresorcinol) and chlorophyll. AP samples resulted richer in phenolic acids (benzoic, 4-vinylsyringol) and flavonoids such as echinatin. Moreover, terpenes like carotenoids (crocetin), terpenoids, and sesquiterpenoids (betulinic acid, oleuropein-aglycone, 5-acetoxyoxachamigrene, lettucenin A) were prevalent, alongside other compounds like (2-oxindol-3-Yl)-acetyl-L-aspartate (amino acid) and tetragalacturonic acid (organic acid). BP samples showcased richness in flavonoids like epicatechin-(2α-7)(4α-8)-epicatechin 3-O-galactoside and cyanidin 3-(feruloyl)(sinapoyl)-triglucoside-5-glucoside, and the terpenoid mascaroside III. Additionally, other compounds like biotinyl-5-adenylate (vitamin) and oleic acid (Fatty acid) were notable in BP samples. 

### 3.4. Bioactive Properties and Phytochemical Content Correlation Matrix

**Figure 7:**
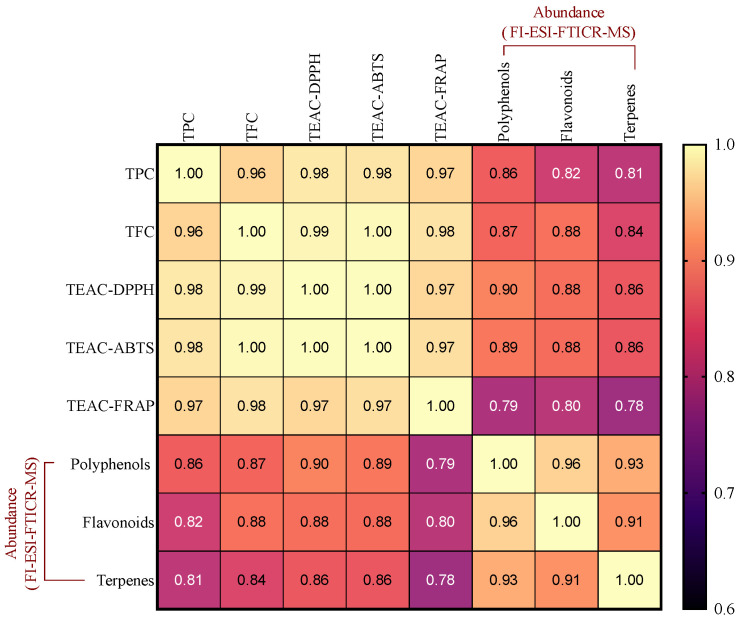
Pearson’s correlation coefficient matrix for the phytochemical content and antioxidant capacity of agro-industrial by-products extracts. Values denote Pearson’s coefficients for each pair of parameters. Lighter gradients denote higher associations. All coefficients were found to be statistically significant (*p* < 0.05).

Potential correlations among all the studied parameters (TPC, TFC, AC, and phytochemical abundance via FI-ESI-FTICR-MS) were examined using Pearson’s correlation test. The resulting correlation matrix is presented in Figure 7. A similar trend between TFC levels and TPC across all samples was observed, as shown in Figure 1, while Figure 2C shows a comparable pattern regarding AC. This observation suggested a potential positive correlation among these parameters, which was further supported by Pearson’s correlation test, where a robust positive correlation with a coefficient r > 0.9 (*p* < 0.05) was noted between TPC, TFC, and AC, indicating that polyphenols present in the sub-products contribute significantly to the antioxidant capacity of these extracts. 

The polyphenols, flavonoids, and terpenes detected via FI-ESI-FTICR-MS also resulted in a strong correlation (*p* < 0.05) with AC. These findings suggest that, within the selected samples in this study, the phenolics and terpenes in the sub-product extracts significantly contributed to the observed antioxidant activities. This underscores the potential of these natural compounds as effective antioxidants and highlights their importance in various applications, including functional foods, nutraceuticals, and pharmaceuticals. Additionally, strong positive correlations among antioxidant assays were also observed. The correlation between DPPH and ABTS assays suggests that the compounds contributing to free radical scavenging activity share comparable hydrophilicity, since the ABTS assay is applicable to both hydrophilic and lipophilic systems, whereas the DPPH assay is constrained to hydrophobic conditions [60]. FRAP was also highly correlated with both DPPH and ABTS. This indicates that compounds capable of scavenging DPPH and ABTS radicals may also exhibit the ability to reduce ferric ions. Furthermore, the dual capability of compounds to scavenge various types of radicals suggests broader applicability and efficacy in combating oxidative stress.

TPC and TFC are commonly positively correlated because many flavonoids are phenolic compounds themselves, and are synthesized through common biosynthetic pathways, often co-occurring in plant tissues [61,62]. Phenolic compounds, abundant in fruits, vegetables, and plant-derived beverages, display potent antioxidant properties, which may significantly contribute to the protective effects of the foods in vivo [63]. Moreover, recent studies have revealed that certain phenolic and flavonoid compounds demonstrate superior antioxidant activity compared with synthetic antioxidants [64,65]. Numerous studies across various plant sources have demonstrated a significant correlation between TPC and TFC levels with AC [66,67,68]. However, the correlation indexes observed in some studies have not been as high as those found in the present work [69,70]. The notably high correlation indexes identified in our study (Figure 7) may suggest that the antioxidant activity is predominantly attributable to compounds of phenolic nature. Nonetheless, some studies have evidenced a lack of correlation between TPC and antioxidant activity [71], indicating that, while the correlation is often observed, it may not be universally applicable across all plant materials. Thus, the potential contribution of non-phenolic compounds to antioxidant capacity should not be overlooked. 

## 4. Conclusions

This comprehensive study underscores the significant potential of agro-industrial residues as sustainable and valuable sources of bioactive compounds, particularly phenolic compounds known for their remarkable antioxidant properties. These findings indicate that, among the various agro-industrial by-products analyzed, cold-brew spent coffee grounds demonstrated significant levels of TPC, TFC, and AC, making them a prime candidate for revalorization in the development of natural preservatives.

The use of advanced analytical techniques, such as FI-ESI-FTICR-MS coupled with univariate and multivariate analysis, allowed for an in-depth exploration of the phytochemical diversity within the residues. Direct injection of the extracts into a high-resolution mass spectrometer revealed over 250 molecular formulas and emphasized the abundance of phenolics, terpenes, alkaloids, and other organic compounds, all with mass errors under 20 ppm. Statistical analysis revealed distinctive patterns among the samples, showcasing unique compounds found in specific by-products.

Strong positive correlations between AC, TPC, TFC, and polyphenols, flavonoids, and terpenes detected via FI-ESI-FTICR-MS demonstrated the significant contribution of these compounds to the antioxidant activities of the extracts.

This multimethodological approach provided a detailed fingerprint of the agro-industrial residues, suggesting their potential for reuse in various applications due to their rich composition as reservoirs of natural antioxidants. The presence of polyphenols, flavonoids, and terpenes underscores the potential of these residues as natural antioxidants, with promising applications in the cosmetic, pharmaceutical, and food supplement industries.

## Figures and Tables

**Table 1:** Summary of compound matches found in positive and negative ESI modes and their classification.

Ionization Mode	ESI+	ESI−	ESI+	ESI−
Phytochemicals	Matched with Databases		Mass Error < 20 ppm	
Alkaloids	28	44	8	15
Carbohydrates	9	16	5	8
Organosulfur and n-containing compounds	13	34	3	14
Polyphenols	97	197	29	71
Terpenes	95	86	34	33
Miscellaneous phytochemicals	19	44	7	6
Other organic compounds	23	29	13	13
Total	284	450	99	160

**Table 2:** Abundant polyphenol peaks identified in agro-industrial by-product extracts via FI-ESI-FTICR-MS analysis. Mean intensity from three replicates (×10^6^).

ESI Mode	Measured *m*/*z*	Error (ppm)	Molecular Formula	Putative Annotation	Subclass	SCG	CBCG	GP	BCP	AP	BP	PP
−	386.1259	11.85	C17H22O10	1-O-Sinapoyl-Beta-D-Glucose	Phenolic acids	7.04	6.40	0.00	20.15	0.00	0.00	6.64
+	362.3189	1.18	C24H42O2	5-Octadecylresorcinol	Alkylresorcinols	17.26	9.79	0.00	8.91	0.00	0.00	2.56
−	488.1390	14.54	C24H24O11	6-O-Acetylglycitin	Flavonoids	73.47	214.33	0.00	0.00	0.71	22.56	5.53
−	374.1364	−0.32	C20H22O7	7-Hydroxymatairesinol	Alkylresorcinols	0.00	0.00	0.00	0.00	7.70	21.06	0.00
−	122.0362	−4.98	C7H6O2	Benzoic Acid	Phenolic acids	8.69	0.00	6.01	3.43	16.58	0.00	11.95
−	216.0436	6.14	C12H8O4	Bergapten	Flavonoids	4.79	6.09	111.24	44.09	5.54	0.00	84.05
+	576.4152	−4.66	C38H56O4	Campesteryl Ferulate	Phenolic acids	74.19	28.29	0.00	221.27	0.46	44.88	7.30
+	302.2595	−4.78	C21H34O	Cardanol	Misc. polyphenols	160.71	141.77	6.57	0.00	0.30	62.93	5.41
+	449.1119	7.89	C21H21O11	Cyanidin-3-Glucoside	Flavonoids	0.00	0.00	9.45	5.11	0.00	23.52	1.13
−	1317.3600	−9.17	C60H69O33	Cyanidin3-(Feruloyl)(Sinapoyl)-Triglucoside-5-Glucoside	Flavonoids	0.00	0.00	0.00	0.00	0.00	70.25	0.00
−	979.2775	5.69	C44H51O25	Cyanidin3-(Sinapoyl)-Diglucoside-5-Glucoside	Flavonoids	0.00	0.00	9.21	0.00	0.69	39.04	2.40
−	491.1259	14.22	C23H23O12	Cyanidin3-O-(6-Acetyl-Glucoside)	Flavonoids	38.10	90.41	0.00	0.00	0.00	11.72	4.17
−	435.0945	4.08	C20H19O11	Delphinidin-3-Arabinoside	Flavonoids	107.76	160.34	0.00	0.00	0.00	0.00	4.18
−	270.0906	5.15	C16H14O4	Echinatin	Flavonoids	0.00	0.00	0.00	3.75	9.70	6.38	0.00
+	706.1948	7.12	C36H34O15	Epicatechin-(2a-7)(4a-8)-Epicatechin 3-O-Galactoside	Flavonoids	0.00	150.64	21.15	0.00	0.00	322.55	0.00
−	594.1696	18.77	C27H30O15	Kaempferol-3-O-Galactoside7-O-Rhamnoside	Flavonoids	0.00	0.00	28.91	0.00	0.00	0.00	0.00
−	534.1010	0.05	C24H22O14	Kaempferol3-O-(6-Malonyl-Glucoside)	Flavonoids	795.20	1443.82	13.68	0.00	3.26	619.05	195.86
+	578.4275	−10.43	C38H58O4	Methylcholestanol Ferulate	Phenolic acids	307.56	159.82	10.51	73.57	0.51	65.69	39.51
−	178.0608	−12.24	C10H10O3	Methyl-p-Coumaric Acid	Phenolic acids	36.25	92.10	4.50	0.00	0.00	5.74	6.94
−	402.1316	0.24	C21H22O8	Nobiletin	Flavonoids	4.23	5.83	0.00	56.73	1.24	24.79	8.28
+	370.2875	0.76	C25H38O2	Nonadecatrienyl-Resorcinol	Alkylresorcinols	668.90	354.55	64.46	60.91	0.00	71.81	142.05
−	378.1295	−5.09	C19H22O8	Oleuropein-Aglycone	Misc. polyphenols	5.02	0.00	47.17	13.78	72.85	81.61	129.33
−	726.2045	5.22	C32H38O19	Pelargonidin-3-Sambubioside-5-Glucoside	Flavonoids	53.27	155.38	0.00	0.00	0.00	8.89	0.00
−	898.1918	−4.31	C45H38O20	Prodelphinidintrimergc-Gc-C	Flavonoids	20.95	25.65	19.06	0.00	0.00	16.62	0.00
−	506.1129	13.63	C23H22O13	Quercetin-3-(6-Acetylglucoside)	Flavonoids	41.99	98.28	0.00	0.00	1.07	11.94	5.11
−	354.1109	1.63	C20H18O6	Sesamin	Lignans	46.18	76.68	0.00	0.00	0.31	0.00	9.96
+	354.1158	15.49	C20H18O6	Sesamin	Lignans	9.56	24.80	13.00	0.00	0.33	64.94	3.22

SCG—spent coffee grounds, CBCG—cold-brew spent coffee grounds, GP—garlic peel, BCP—black carrot pomace, PP—pineapple peel, AP—apple pomace, BP—beetroot pomace.

**Table 3:** Abundant terpene peaks identified in agro-industrial by-product extracts via FI-ESI-FTICR-MS analysis. Mean intensity from three replicates (×10^6^).

ESI Mode	Measured *m*/*z*	Error (ppm)	Molecular Formula	Putative Annotation	Subclass	SCG	CBCG	GP	BCP	AP	BP	PP
+	540.3652	−1.86	C30H52O8	(+)-Longilene peroxide	Triterpenoids	19.33	30.84	4.53	99.62	1.18	13.90	7.18
−	306.2533	−8.54	C20H34O2	Akhdardiol	Diterpenoids	142.51	93.53	40.33	103.56	0.00	0.00	1.82
+	306.2568	3.13	C20H34O2	Akhdardiol	Diterpenoids	0.00	8.15	50.95	118.74	0.31	0.00	0.00
+	288.2444	−3.24	C20H32O	Akhdarenol	Diterpenoids	135.62	54.79	2.53	0.00	0.00	271.64	36.03
−	488.3593	18.62	C30H48O5	Asiatic Acid	Triterpenoids	0.00	0.00	0.00	117.34	0.00	0.00	5.96
−	456.3632	6.34	C30H48O3	Betulinic Acid	Triterpenoids	0.00	0.00	0.00	11.25	4.86	0.00	0.00
+	596.4879	12.42	C39H64O4	Cafestolstearate	Diterpenoids	272.83	701.28	119.11	301.06	0.44	0.00	13.32
+	586.4168	−11.12	C36H58O6	Caffarolide B	Diterpenoids	18.18	24.43	5.62	0.00	0.00	0.00	24.43
	594.3925	0.73	C37H54O6	Caffarolide F	Diterpenoids	22.15	35.24	9.21	42.11	0.00	0.00	0.00
+	608.4037	−6.59	C38H56O6	Caffarolide G	Diterpenoids	96.63	207.68	7.84	250.35	0.00	203.23	0.00
−	564.3921	−8.26	C40H52O2	Canthaxanthin	Carotenoids	21.27	11.66	17.39	0.00	0.00	0.00	4.77
+	424.3662	−10.07	C30H48O	Cycloeucalenone	Triterpenoids	155.94	38.04	9.16	0.00	0.00	0.00	0.00
−	414.3137	0.68	C27H42O3	Diosgenin	Saponins	70.78	28.91	0.00	0.00	0.00	0.00	15.57
+	584.4227	−0.34	C40H56O3	Flavoxanthin	Carotenoids	292.32	79.93	67.99	32.03	0.49	29.53	30.97
−	360.1190	−5.19	C19H20O7	Gibberellin A8-Catabolite	Diterpenoids	0.00	0.00	0.00	0.00	2.33	0.00	3.29
+	456.1494	−3.86	C22H33BrO5	Isoparguerol	Diterpenoids	0.00	0.00	0.00	0.00	0.52	4.29	7.12
−	240.0813	11.10	C15H12O3	Lettucenin A	Sesquiterpenoids	0.00	0.00	0.00	11.38	10.27	0.00	0.00
−	684.2740	−6.05	C36H44O13	MascarosideIII	Diterpenoids	13.83	36.49	0.00	0.00	6.95	232.52	6.49
+	152.1192	−6.27	C10H16O	Myrcenol	Monoterpenoids	0.00	0.00	0.00	8.37	0.00	98.79	0.00
+	496.0853	5.92	C20H34Br2O4	Neoirietetraol	Diterpenoids	12.26	40.70	0.00	8.46	0.38	44.40	0.00
+	600.4195	2.76	C40H56O4	Neoxanthin	Carotenoids	252.21	160.91	46.02	160.39	0.44	14.74	40.43
−	600.4260	13.48	C40H56O4	Neoxanthin	Carotenoids	8.12	0.00	21.85	17.03	0.00	0.00	6.85
+	936.4179	−2.46	C43H68O22	Rebaudioside F	Diterpenoids	0.00	0.00	0.00	0.00	0.00	0.00	14.32
−	936.4312	11.71	C43H68O22	Rebaudioside F	Diterpenoids	47.28	35.85	0.00	0.00	0.00	4.43	0.00
+	795.4651	15.10	C42H67O14	Soyasaponin III	Saponins	370.27	382.64	0.00	48.25	12.00	214.64	0.00
+	804.3780	0.08	C38H60O18	Stevioside	Diterpenoids	47.23	26.60	0.00	30.66	0.63	0.00	26.41
+	592.4430	−10.32	C39H60O4	Stigmastanol Ferulate	Triterpenoids	175.27	1674.56	155.30	293.40	0.83	623.35	4.65
−	592.4508	2.69	C39H60O4	Stigmastanol Ferulate	Triterpenoids	62.48	56.20	15.32	17.02	0.00	3.12	4.33

SCG—spent coffee grounds, CBCG—cold-brew spent coffee grounds, GP—garlic peel, BCP—black carrot pomace, PP—pineapple peel, AP—apple pomace, BP—beetroot pomace.

## Data Availability

The data presented in this study are available on request from the corresponding author.

## Supplementary Materials

The following supporting information can be downloaded at: https://www.mdpi.com/article/10.3390/antiox13080925/s1, Table S1: Standard calibration curves; Table S2: Phytochemical content and antioxidant capacity in agro-industrial by-products extracts (summary); Table S3: Identified peaks in by-product extracts via FI-ESI-FTICR-MS analysis in positive ESI mode: mean intensity from three replicates (×10^6^); Table S4: Identified peaks in by-product extracts via FI-ESI-FTICR-MS analysis in negative ESI mode: mean intensity from three replicates (×10^6^); Figure S1: PLS-DA model validation in (A) positive and (B) negative ESI modes datasets by permutation tests based on separation distance. The *p* value based on permutation is *p* < 0.001 (0/1000).
