# Supplementary material for: Exploring Agro-Industrial By-Products: Phenolic Content, Antioxidant Capacity, and Phytochemical Profiling via FI-ESI-FTICR-MS Untargeted Analysis
Source: Antioxidants (Basel). 2024 Jul 30;13(8):925. doi: 10.3390/antiox13080925 (PMC11351152; doi:10.3390/antiox13080925)
Supplement: Supplementary file 1 [file antioxidants-13-00925-s001.zip › antioxidants-3118798-supplementary.pdf]

SUPPLEMENTARY MATERIAL

Exploring Agro-Industrial By-Products: Phenolic Content, Antioxidant Capacity and Phytochemical Profiling Via FI-ESI-FTICR-MS Untargeted Analysis

Itzel Yoali Hernández-Montesinos [1], David Fernando Carreón-Delgado [1], Oxana Lazo-Zamalloa [1], Lilia Tapia-López [1], Minerva Rosas-Morales [1], Carlos Enrique Ochoa-Velasco [2], Paola Hernández-Carranza [2], Yair Cruz-Narváez [3] and Carolina Ramírez-López [1,*]

[1] Centro de Investigación en Biotecnología Aplicada, Instituto Politécnico Nacional, Carretera Estatal Santa Inés Tecuexcomac-Tepetitla, km 1.5, Tepetitla de Lardizábal, Tlaxcala 90700, Mexico.

[2] Facultad de Ciencias Químicas, Benemérita Universidad Autónoma de Puebla, 4 Sur 104, Centro Histórico, Puebla 72000, Mexico.

[3] Laboratorio de Posgrado de Operaciones Unitarias, Escuela Superior de Ingeniería Química e Industrias Extractivas, Instituto Politécnico Nacional, Av. Instituto Politécnico Nacional, Lindavista, Gustavo A. Madero, Ciudad de México 07700, Mexico.

* **Correspondence**: caramirezl@ipn.mx (C.R-L.); Tel.: +52-2223237866 (C.R.-L.)

Itzel Yoali Hernández-Montesinos, Instituto Politécnico Nacional, Centro de Investigación en Biotecnología Aplicada, Carretera Estatal Santa Inés Tecuexcomac-Tepetitla, km 1.5, Tepetitla de Lardizábal, Tlaxcala 90700, Mexico. E-mail: ihernandezm1308@alumno.ipn.mx

David Fernando Carreón-Delgado, Instituto Politécnico Nacional, Centro de Investigación en Biotecnología Aplicada, Carretera Estatal Santa Inés Tecuexcomac-Tepetitla, km 1.5, Tepetitla de Lardizábal, Tlaxcala 90700, Mexico.. E-mail: dcarreond1300@alumno.ipn.mx

Table S1. Standard calibration curves

Total Phenolic Content

[C] Gallic Acid mg/mL	Absorbance at 765 nm		
0	0	0	0
0.05	0.165	0.167	0.171
0.1	0.286	0.291	0.294
0.15	0.423	0.425	0.421
0.2	0.584	0.584	0.573
0.25	0.745	0.75	0.73
0.3	0.862	0.86	0.871
0.35	0.959	0.956	0.976

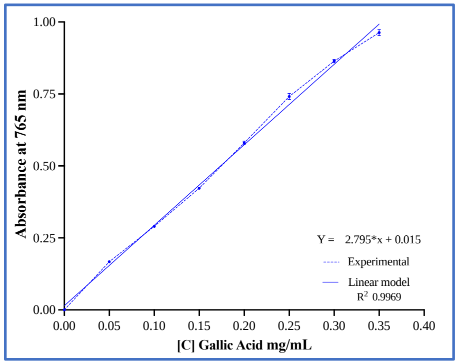

Total Flavonoid Content

[C] Quercetin mg/mL	Absorbance at 510 nm		
0	0	0	0
0.1	0.105	0.114	0.102
0.2	0.269	0.285	0.301
0.3	0.422	0.394	0.402
0.4	0.551	0.568	0.569
0.5	0.714	0.726	0.749
0.6	0.897	0.918	0.889
0.7	1.016	1.004	1.003

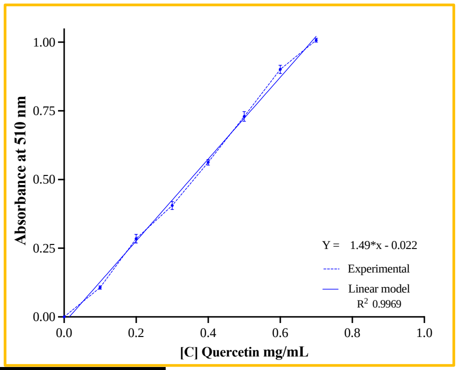

Trolox antioxidant capacity calibration curve - DPPH assay

[C] Trolox µM	DPPH radical scavenging capacity (%)			
400	96.76	96.43	94.16	95.78
200	94.43	92.48	93.46	92.81
100	85.54	84.24	86.19	85.21
50	68.83	70.13	67.86	70.13
25	36.69	39.93	40.25	40.58
12.5	23.11	24.36	25.74	23.71
6.25	19.49	15.92	14.3	14.63
3.125	8.33	8.01	7.36	6.71
1.5625	5.84	3.24	4.54	2.6
0	0	0	0	0

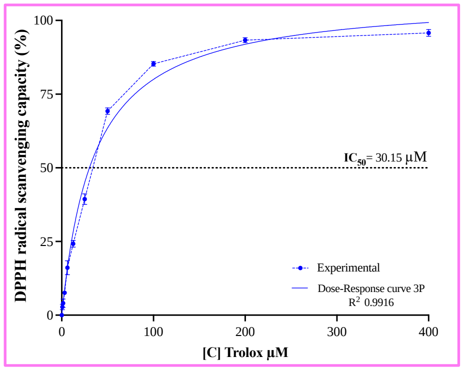

Trolox antioxidant capacity calibration curve - ABTS assay

[C] Trolox μM	ABTS radical scavenging capacity (%)			
400	96.76	96.43	94.16	95.78
200	94.43	92.48	93.46	92.81
100	85.54	84.24	86.19	85.21
50	68.83	70.13	67.86	70.13
25	36.69	39.93	40.25	40.58
12.5	23.11	24.36	25.74	23.71
6.25	19.49	15.92	14.3	14.63
3.125	8.33	8.01	7.36	6.71
1.5625	5.84	3.24	4.54	2.6
0	0	0	0	0

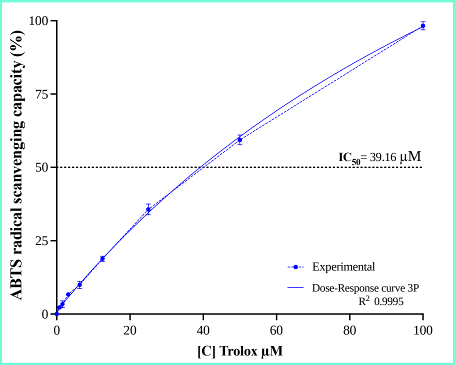

Trolox antioxidant capacity calibration curve - FRAP assay

[C] Trolox μM	Absorbance at 593 nm			
100	0.643	0.641	0.679	0.633
50	0.368	0.374	0.389	0.382
25	0.161	0.212	0.197	0.197
12.5	0.089	0.113	0.114	0.098
6.25	0.051	0.066	0.07	0.047
3.125	0.025	0.039	0.032	0.021
1.5625	0.01	0.015	0.013	0.01
0.78125	0.002	0.004	0.009	0.005
0	0	0	0	0

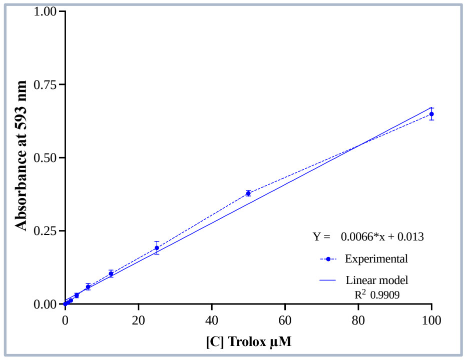

Table S2. Phytochemical content and Antioxidant capacity in Agro-Industrial By-Products extracts (Summary)

Phytochemical content and Antioxidant capacity		SCG	CBCG	GP	BCP	AP	BP	PP
**Phenolic and Flavonoid Content**								
TPC	mgGAE/g	95.64 ± 0.84 [a]	159.94 ± 0.9 [b]	53.35 ± 0.33 [c]	26.83 ± 0.17 [d]	9.11 ± 0.06 [e]	7.17 ± 0.13 [f]	22.96 ± 0.12 [g]
TFC	mgQE/g	47.93 ± 0.79 [a]	128.37 ± 0.5 [b]	20.52 ± 2.14 [c]	15.26 ± 0.32 [d]	6.44 ± 0.09 [e]	5.76 ± 0.42 [e]	15.35 ± 0.12 [d]
**Antioxidant Capacity**								
TEAC-DPPH	IC50 mg/mL	0.079 ± 0.001 [a]	0.036 ± 0.001 [b]	0.275 ± 0.009 [c]	0.340 ± 0.008 [d]	4.833 ± 0.013 [e]	5.526 ± 0.009 [f]	0.678 ± 0.015 [g]
	(umolTx/g)	394.28 ± 5.34 [a]	863.00 ± 6.89 [b]	112.88 ± 3.68 [c]	91.33 ± 2.11 [d]	6.43 ± 0.40 [e]	5.61 ± 0.16 [e]	45.88 ± 2.98 [f]
TEAC-ABTS	IC50 mg/mL	0.094 ± 0.001 [a]	0.04 ± 0.001 [b]	0.284 ± 0.015 [c]	0.349 ± 0.005 [d]	5.683 ± 0.003 [e]	5.450 ± 0.011 [f]	0.692 ± 0.012 [g]
	(umolTx/g)	416.54 ± 4.6 [a]	990.64 ± 8.53 [b]	138.01 ± 7.31 [c]	112.58 ± 8.17 [d]	6.95 ± 0.81 [e]	7.19 ± 0.26 [e]	56.72 ± 3.51 [f]
FRAP-Tx	(umolTx/g)	146.59 ± 0.22 [a]	366.71 ± 0.47 [b]	130.59 ± 0.61 [c]	85.88 ± 3.83 [d]	25.99 ± 0.21 [e]	22.49 ± 1.45 [e]	82.64 ± 3.41 [d]

ANOVA and post hoc Tukey test results (depicted as lower-case letters) assessing the difference among residues extracts: Different letters indicate significant differences (α = 0.05).

Table S3. Identified peaks in by-product extracts via FI-ESI-FTICR-MS analysis in positive ESI mode: Mean intensity from three replicates (x10[6]).

Measured m/z	Theoretical m/z	Mass error < 20 ppm	Molecular Formula	Putative Annotation	GP	BP	SCG	CBCG	AP	PP	BCP
288.99728	288.99497	8.01	C10H12BrNO4	2-amino-3-(3-bromo-5-hydroxy-4-methoxyphenyl)propanoic acid	0.00	0.00	54.90	7.88	0.00	0.00	0.00
152.11916	152.12012	-6.27	C10H16O	Myrcenol	0.00	98.79	0.00	0.00	0.00	8.37	0.00
184.14631	184.14633	-0.13	C11H20O2	Linalool Oxide	0.00	0.00	67.94	7.97	0.00	0.00	0.00
304.07193	304.06954	7.88	C14H12N2O6	(2-Oxindol-3-Yl) Acetyl-L-Aspartate	0.00	66.04	0.00	0.00	0.00	0.00	0.00
311.10331	311.10050	9.02	C14H17NO7	Dhurrin	0.00	0.00	0.00	0.00	0.32	6.93	0.00
190.17544	190.17215	17.28	C14H22	Patchoulen-Beta	9.82	41.89	0.00	0.00	0.00	0.00	0.00
523.82017	523.81057	18.32	C15H11Br3O6	(3-Bromo-5-hydroxy-4-methoxyphenyl)(2,3-dibromo-4,5-dihydroxyphenyl)acetic acid	2.92	0.00	0.00	0.00	0.00	0.00	0.00
509.82253	509.83131	-17.23	C15H13Br3O5	3,4-dibromo-5-[2-bromo-3,4-dihydroxy-6-(methoxymethyl)benzyl]benzene-1,2-diol	0.00	0.00	0.00	8.79	0.00	0.00	3.59
350.05962	350.06482	-14.85	C15H24BrClO2	a-bisabolene	2.36	0.00	0.00	0.00	0.00	16.12	0.00
272.10148	272.10486	-12.41	C16H16O4	1 3-Cis-Tetrahydroxyphenylindan	0.00	0.00	0.00	0.00	0.00	0.00	34.27
306.10845	306.11034	-6.17	C16H18O6	1-Naphthol Glucoside	0.00	15.50	0.00	0.00	0.34	0.00	0.00
316.18723	316.18859	-4.29	C16H28O6	Geranyl Beta-D-Glucopyranoside	7.47	0.00	0.00	0.00	0.00	10.58	8.71
298.08837	298.08412	14.25	C17H14O5	Apigenin 7 4-Dimethylether	0.00	11.54	0.00	0.00	0.00	0.00	0.00
375.13684	375.13046	17.01	C17H19N4O6	Riboflavin	0.00	0.00	0.00	0.00	0.00	9.33	0.00
368.10987	368.11073	-2.34	C17H20O9	3-Feruloylquinic Acid	0.00	0.00	0.00	8.19	0.00	18.14	0.00
392.07201	392.07538	-8.62	C17H26BrClO3	5-acetoxyoxachamigrene	0.00	21.99	0.00	74.21	0.00	0.00	0.00
426.17753	426.17373	8.93	C17H30O12	4-(2-Methylbutanoyl)Sucrose	39.08	0.00	0.00	0.00	0.00	0.00	0.00
418.14373	418.14751	-9.04	C18H26O11	Oleosidedimethylester	15.69	12.39	0.00	0.00	0.00	13.34	0.00
297.24371	297.24297	2.49	C18H33O3	Ricinoleate	3.39	99.13	440.22	733.32	0.30	17.98	8.90
282.25780	282.25588	6.81	C18H34O2	Oleic Acid	0.00	656.48	45.52	8.19	0.36	108.54	0.00
329.16732	329.16271	14.02	C19H23NO4	Reticuline	0.00	0.00	0.00	0.00	0.38	12.24	0.00

354.11582	354.11034	15.49	C20H18O6	Sesamin	13.00	64.94	9.56	24.80	0.33	0.00	3.22
353.12447	353.12632	-5.26	C20H19NO5	Chelidonine	0.00	16.61	0.00	0.00	0.00	5.66	0.00
326.14894	326.15181	-8.81	C20H22O4	Crocetin	0.00	30.88	0.00	0.00	0.00	0.00	0.00
424.14324	424.13695	14.83	C20H24O10	Salicortin	2.61	0.00	0.00	0.00	0.00	0.00	0.00
572.13031	572.13286	-4.45	C20H27N7O9P S	Biotinyl-5-Adenylate	0.00	879.19	0.00	31.00	0.00	66.06	0.00
318.22272	318.21949	10.12	C20H30O3	Steviol	14.95	0.00	0.00	0.00	0.00	0.00	3.11
381.21493	381.21514	-0.53	C20H31NO6	Echiumine	33.57	0.00	0.00	0.00	0.31	0.00	0.00
288.24438	288.24532	-3.24	C20H32O	Akhdarenol	2.53	271.64	135.62	54.79	0.00	0.00	36.03
336.23338	336.23006	9.88	C20H32O4	Luputrione	6.61	0.00	0.00	0.00	0.00	0.00	0.00
496.08532	496.08238	5.92	C20H34Br2O4	Neoirietetraol	0.00	44.40	12.26	40.70	0.38	8.46	0.00
306.25684	306.25588	3.13	C20H34O2	Akhdardiol	50.95	0.00	0.00	8.15	0.31	118.74	0.00
453.21131	453.21710	-12.78	C20H39O7P2	Phytyl Diphosphate	0.00	11.58	0.00	0.00	0.34	0.00	0.00
446.09039	446.08491	12.29	C21H18O11	Apigenin7-O-Glucuronide	0.00	0.00	0.00	0.00	0.00	0.00	4.01
449.11193	449.10839	7.89	C21H21O11	Cyanidin-3-Glucoside	9.45	23.52	0.00	0.00	0.00	5.11	1.13
356.15908	356.16237	-9.24	C21H24O5	Gingerenonea	0.00	0.00	0.00	0.00	0.00	0.00	7.08
356.18017	356.18618	-16.88	C21H26NO4	Menisperine	0.00	0.00	0.00	0.00	0.00	0.00	2.32
358.19561	358.20183	-17.37	C21H28NO4	(S)-Laudanosine	0.00	0.00	0.00	0.00	0.00	0.00	2.68
348.23397	348.23006	11.24	C21H32O4	Trans-Isohumulone	2.89	0.00	0.00	0.00	0.00	0.00	0.00
302.25952	302.26097	-4.78	C21H34O	Cardanol	6.57	62.93	160.71	141.77	0.30	0.00	5.41
478.10586	478.11113	-11.02	C22H22O12	Isorhamnetin3-O-Galactoside	0.00	0.00	0.00	9.56	0.00	0.00	0.00
463.11544	463.12404	-18.57	C22H23O11	Malvidin3-O-Arabinoside	0.00	0.00	0.00	0.00	0.00	7.36	0.00
432.13785	432.14203	-9.69	C22H24O9	3-Methoxynobiletin	10.19	20.34	0.00	0.00	0.00	0.00	0.00
418.15815	418.16277	-11.04	C22H26O8	Syringaresinol	0.00	11.81	0.00	0.00	0.00	0.00	0.00
528.09879	528.10345	-8.84	C23H25ClO12	Malvidin3-O-Galactoside	0.00	0.00	0.00	0.00	0.00	0.00	2.41
386.20622	386.20932	-8.03	C23H30O5	Mascarosidev	3.49	0.00	0.00	0.00	0.35	0.00	0.00
722.13311	722.13892	-8.04	C24H34O25	Tetragalacturonic Acid	0.00	212.87	0.00	0.00	0.00	0.00	0.00
362.31891	362.31848	1.18	C24H42O2	5-Octadecylresorcinol	0.00	0.00	17.26	9.79	0.00	8.91	2.56

666.23037	666.22186	12.78	C24H42O21	Stachyose	120.21	46.92	0.00	0.00	0.00	0.00	0.00
532.11238	532.12169	-17.50	C25H24O13	6-O-Malonylglycitin	3.06	0.00	0.00	0.00	0.00	0.00	0.00
370.28746	370.28718	0.76	C25H38O2	Nonadecatrienylresorcinol	64.46	71.81	668.90	354.55	0.00	60.91	142.05
414.27546	414.27701	-3.75	C26H38O4	Lupulone	3.09	0.00	36.42	17.10	0.00	0.00	3.76
630.12597	630.13515	-14.56	C27H31ClO15	Pelargonidin3 5-O-Diglucoside	0.00	0.00	0.00	0.00	0.00	0.00	4.76
579.17100	579.17138	-0.66	C27H31O14	Pelargonidin3-O-Rutinoside	0.00	0.00	0.00	0.00	0.00	0.00	3.12
394.29273	394.28718	14.07	C27H38O2	Sargaquinone	10.62	0.00	0.00	0.00	0.37	5.32	0.00
414.31368	414.31340	0.68	C27H42O3	Diosgenin	0.00	0.00	70.78	28.91	0.00	0.00	15.57
414.33997	414.33720	6.67	C27H44NO2	Solasodine	11.78	52.90	447.74	182.73	0.00	61.61	52.81
447.33984	447.33486	11.14	C27H45NO4	Esculeogeninb	0.00	0.00	0.00	6.55	0.00	0.00	0.00
446.34058	446.33961	2.16	C28H46O4	3-Dehydroteasterone	3.70	24.19	95.01	128.55	0.00	9.66	5.78
448.36411	448.35526	19.74	C28H48O4	Teasterone	15.60	0.00	0.00	18.57	0.00	30.02	9.97
450.36532	450.37091	-12.42	C28H50O4	6-Hydroxyteasterone	5.00	7.37	15.16	0.00	0.37	109.19	8.90
436.32707	436.33413	-16.18	C30H44O2	Demethylphylloquinone	2.65	33.12	44.23	8.78	0.00	8.39	10.17
452.33275	452.32905	8.18	C30H44O3	Zizyberenalic Acid	9.33	0.00	0.00	0.00	0.00	16.53	5.02
424.36624	424.37052	-10.07	C30H48O	Cycloeucalenone	9.16	0.00	155.94	38.04	0.00	0.00	0.00
440.36479	440.36543	-1.46	C30H48O2	Betulinic Aldehyde	21.31	0.00	0.00	6.89	0.00	0.00	1.14
457.36522	457.36817	-6.45	C30H49O3	(3beta,9beta)-4alpha-Demethyl-4alpha-Carboxy-9,19-Cyclolanost-3-Ol	3.02	92.75	0.00	0.00	0.59	6.99	0.00
540.36521	540.36622	-1.86	C30H52O8	(+)-Longilene peroxide	4.53	13.90	19.33	30.84	1.18	99.62	7.18
452.37292	452.36543	16.56	C31H48O2	Phylloquinol	33.01	0.00	0.00	0.00	0.00	0.00	0.00
650.34736	650.35136	-6.15	C31H54O14	3-Acetyl-3-Dodecanoyl-4-(3-Methylbutanoyl)Sucrose	7.20	0.00	0.00	0.00	0.00	0.00	0.00
460.43572	460.42803	16.70	C31H56O2	5-Pentacosylresorcinol	3.12	0.00	0.00	0.00	0.00	0.00	0.00
641.32773	641.31732	16.24	C32H49O13	Steviolbioside	0.00	0.00	0.00	0.00	0.00	0.00	3.72
593.45811	593.45162	10.94	C32H59N5O5	Galaxamide	13.39	14.94	31.39	69.15	0.00	0.00	0.00
576.39254	576.39003	4.37	C33H54NO7	Solasodine 3-O-Beta-D-Glucoside	16.25	0.00	15.05	10.50	0.00	21.66	12.89
678.38805	678.38266	7.95	C33H58O14	3-Dodecanoyl-3-Isobutanoyl-4-(3-Methylbutanoyl)Sucrose	12.15	0.00	51.61	0.00	0.00	217.48	0.00

692.41056	692.39831	17.69	C34H60O14	3-Dodecanoyl-3,4-Di(3-Methylbutanoyl)Sucrose	7.21	0.00	0.00	0.00	0.00	0.00	0.00
706.19480	706.18977	7.12	C36H34O15	Epicatechin-(2a-7)(4a-8)-Epicatechin 3-O-Galactoside	21.15	322.55	0.00	150.64	0.00	0.00	0.00
552.42539	552.41786	13.64	C36H56O4	Kahweolpalmitate	15.50	13.35	14.11	40.71	0.00	289.18	11.90
586.41682	586.42334	-11.12	C36H58O6	Caffarolide B	5.62	0.00	18.18	24.43	0.00	0.00	24.43
673.30887	673.29993	13.28	C37H43N3O9	Triferuloyl Spermidine	16.81	0.00	0.00	0.00	0.00	0.00	0.00
594.39247	594.39204	0.73	C37H54O6	Caffarolidef	9.21	0.00	22.15	35.24	0.00	42.11	0.00
576.41518	576.41786	-4.66	C38H56O4	Campesteryl Ferulate	0.00	44.88	74.19	28.29	0.46	221.27	7.30
608.40368	608.40769	-6.59	C38H56O6	Caffarolideg	7.84	203.23	96.63	207.68	0.00	250.35	0.00
578.42748	578.43351	-10.43	C38H58O4	Methylcholestanol Ferulate	10.51	65.69	307.56	159.82	0.51	73.57	39.51
804.37803	804.37797	0.08	C38H60O18	Stevioside	0.00	0.00	47.23	26.60	0.63	30.66	26.41
602.39441	602.39712	-4.50	C39H54O5	Ursolic Acid	9.54	11.88	0.00	0.00	0.00	15.89	0.00
634.37722	634.38695	-15.35	C39H54O7	3-O-Trans-P-Coumaroyltormentic Acid	0.00	0.00	31.18	0.00	0.00	13.67	0.00
592.44305	592.44916	-10.32	C39H60O4	Stigmastanol Ferulate	155.30	623.35	175.27	1674.56	0.83	293.40	4.65
596.48787	596.48046	12.42	C39H64O4	Cafestolstearate	119.11	0.00	272.83	701.28	0.44	301.06	13.32
550.42362	550.41747	11.19	C40H54O	Anhydrolutein I	26.80	0.00	0.00	7.72	0.00	7.66	0.00
584.42275	584.42295	-0.34	C40H56O3	Flavoxanthin	67.99	29.53	292.32	79.93	0.49	32.03	30.97
600.41952	600.41786	2.76	C40H56O4	Neoxanthin	46.02	14.74	252.21	160.91	0.44	160.39	40.43
795.46509	795.45308	15.10	C42H67O14	Soyasaponin Iii	0.00	214.64	370.27	382.64	12.00	48.25	0.00
936.41792	936.42022	-2.46	C43H68O22	Rebaudioside F	0.00	0.00	0.00	0.00	0.00	0.00	14.32
892.53630	892.53531	1.11	C55H72MgN4O5	Chlorophyll	92.61	0.00	174.24	481.55	0.64	115.53	222.95
130.09734	130.09938	-15.70	C7H14O2	Isoamylacetate	0.00	101.34	0.00	15.48	0.28	89.23	0.00
137.04797	137.04768	2.14	C7H7NO2	Trigonelline	0.00	0.00	136.47	246.05	0.37	0.00	0.00
169.07528	169.07389	8.19	C8H11NO3	Pyridoxine	3.42	0.00	0.00	0.00	0.00	0.00	0.00
247.04366	247.04838	-19.12	C8H12N2O5P	Pyridoxamine 5-Phosphate	0.00	0.00	0.00	0.00	0.36	0.00	0.00
341.86089	341.85609	14.06	C8H8Br2O3S	2,3-dibromo-4,5-dihydroxybenzyl methyl sulfoxide	0.00	0.00	0.00	0.00	0.40	0.00	0.00

Table S4. Identified peaks in by-product extracts via FI-ESI-FTICR-MS analysis in negative ESI mode: Mean intensity from three replicates (x10^6).

Measured m/z	Theoretical m/z	Mass error < 20 ppm	Molecular Formula	Putative Annotation	GP	BP	SCG	CBCG	AP	PP	BCP
178.06081	178.06299	-12.24	C10H10O3	Methylp-Coumaric Acid	4.50	5.74	36.25	92.10	0.00	6.94	0.00
241.05510	241.05864	-14.66	C10H11NO6	2 4-Dihydroxy-7 8-Dimethoxy-1 4-Benzoxazin-3-One	0.00	21.22	0.00	0.00	0.00	1.40	0.00
177.10134	177.10279	-8.17	C10H13N2O	Serotonin	18.78	0.00	0.00	0.00	0.00	0.00	176.56
134.10843	134.10955	-8.33	C10H14	Cymene-P	0.00	0.00	6.04	0.00	0.61	3.18	0.00
188.04038	188.04082	-2.35	C10H8N2S	3-(Isothiocyanatomethyl)-1h-Indole	4.56	0.00	0.00	0.00	0.00	0.00	78.16
190.05221	190.05042	9.45	C10H8NO3	2-Oxoindole-3-Acetate	7.32	8.35	0.00	0.00	0.00	2.40	3.61
209.04548	209.04500	2.31	C10H9O5	5-Hydroxyferulate	0.00	0.00	39.15	52.78	0.00	0.00	0.00
324.06642	324.06361	8.69	C11H20N2O3S3	Erucinn-Acetyl-Cysteine	0.00	16.33	0.00	0.00	0.33	7.88	0.00
406.03726	406.03002	17.82	C11H20NO9S3	Glucoiberverin	0.00	9.90	0.00	0.00	0.00	0.00	0.00
182.08172	182.08440	-14.72	C12H10N2	Harman	0.00	0.00	0.00	0.00	0.00	0.00	7.27
222.09265	222.08921	15.49	C12H14O4	Apiole	0.00	0.00	0.00	0.00	0.32	0.00	0.00
370.07018	370.07474	-12.32	C12H18O13	Digalacturonic Acid	0.00	34.50	0.00	0.00	0.00	0.00	0.00
399.05826	399.06271	-11.15	C12H20N2O9PS	(R)-4-Phosphopantothenoyl-L-Cysteine	0.00	0.00	40.44	110.60	0.00	0.00	0.00
386.06319	386.05795	13.57	C12H20NO9S2	Glucobrassicanapin	0.00	0.00	0.00	0.00	0.00	2.14	0.00
342.11914	342.11621	8.57	C12H22O11	Lactose	0.00	10.29	3.77	4.90	3.17	3.46	0.00
216.04359	216.04226	6.14	C12H8O4	Bergapten	111.24	0.00	4.79	6.09	5.54	84.05	44.09
212.09306	212.09496	-8.98	C13H12N2O	Harmine	0.00	0.00	0.00	0.00	0.00	0.00	6.80
280.05316	280.05830	-18.37	C13H12O7	P-Coumaroylmalic Acid	0.00	0.00	0.00	0.00	0.39	0.00	0.00
314.09396	314.10017	-19.77	C14H18O8	Methylsalicylate-2-O-Beta-D-Glucoside	0.00	0.00	0.00	0.00	5.48	4.69	0.00
267.19343	267.19468	-4.66	C14H25N3O2	N-Caprylhistidinol	0.00	0.00	30.35	13.06	0.57	0.00	0.00
255.06298	255.06573	-10.81	C15H11O4	(2s)-Pinocembrin	0.00	0.00	5.58	0.00	0.00	0.00	0.00
240.08131	240.07864	11.10	C15H12O3	Lettucenin A	0.00	0.00	0.00	0.00	10.27	0.00	11.38

242.09710	242.09429	11.58	C15H14O3	4-Vinylsyringol	0.00	0.00	0.00	0.00	0.00	0.00	29.75
322.06963	322.06887	2.36	C15H14O8	(2r,3s,4s)-Leucodelphinidin	5.97	0.00	0.00	0.00	1.96	0.00	0.00
348.10603	348.10698	-2.76	C15H16N4O6	Histidine-Betaxanthin	0.00	0.00	0.00	0.00	0.42	0.00	0.00
292.04352	292.04628	-9.45	C15H17BrO	4-Bromo-1,10-epoxylaur-11-ene	0.00	0.00	0.00	0.00	0.87	3.07	6.64
342.09403	342.09508	-3.06	C15H18O9	Caffeic Acid 4-O-Glucoside	0.00	0.00	0.00	0.00	0.46	0.00	0.00
240.21373	240.20893	19.97	C15H28O2	Menthylisovalerate	0.00	0.00	0.00	0.00	0.38	0.00	8.28
317.06503	317.06613	-3.45	C16H13O7	Petunidin	0.00	0.00	0.00	0.00	0.00	0.00	4.25
270.09060	270.08921	5.15	C16H14O4	Echinatin	0.00	6.38	0.00	0.00	9.70	0.00	3.75
319.07877	319.08178	-9.42	C16H15O7	Trans-5-O-(4-Coumaroyl)Shikimate	0.00	0.00	0.00	0.00	0.00	1.38	3.59
272.11003	272.10486	18.99	C16H16O4	1 3-Cis-Tetrahydroxyphenylindan	0.00	0.00	0.00	0.00	0.00	0.00	8.56
336.08410	336.08452	-1.26	C16H16O8	5-O-Caffeoylshikimic Acid	0.00	0.00	22.66	74.64	0.38	0.00	0.00
286.13249	286.13174	2.60	C16H18N2O3	Tangutoride	0.00	0.00	0.00	0.00	0.00	0.00	8.74
342.12690	342.13147	-13.34	C16H22O8	Coniferin	0.00	3.53	0.00	0.00	2.07	2.84	0.00
314.07913	314.07904	0.30	C17H14O6	Cirsimaritin	0.00	0.00	0.00	0.00	3.21	2.18	0.00
330.07271	330.07395	-3.75	C17H14O7	3 7-Dimethylquercetin	0.00	0.00	0.00	0.00	0.00	2.80	0.00
346.11719	346.11649	2.04	C17H18N2O6	Dopamine-Betaxanthin	0.00	0.00	0.00	14.95	0.00	4.34	0.00
270.12527	270.12559	-1.20	C17H18O3	Conferol B	0.00	0.00	0.00	0.00	0.38	0.00	0.00
317.12051	317.12632	-18.32	C17H19NO5	Haemanthidine	0.00	8.14	0.00	0.00	0.00	1.84	0.00
368.10427	368.11073	-17.56	C17H20O9	3-Feruloylquinic Acid	0.00	0.00	12.82	9.86	0.00	0.00	0.00
386.12587	386.12130	11.85	C17H22O10	1-O-Sinapoyl-Beta-D-Glucose	0.00	0.00	7.04	6.40	0.00	6.64	20.15
290.17567	290.17562	0.17	C17H24NO3	Littorine	0.00	0.00	0.00	0.00	0.00	0.00	13.25
372.13536	372.14203	-17.92	C17H24O9	Syringin	0.00	0.00	0.00	0.00	0.54	1.71	0.00
300.23296	300.23006	9.65	C17H32O4	Gingerol	0.00	0.00	0.00	0.00	0.00	0.00	3.55
384.06330	384.05937	10.24	C18H12N2O8	Betanidin Quinone	0.00	35.70	0.00	0.00	0.00	3.70	0.00
388.08852	388.09067	-5.54	C18H16N2O8	Betanidin	0.00	20.70	0.00	0.00	0.60	2.51	0.00
328.09653	328.09469	5.60	C18H16O6	Betagarin	0.00	0.00	0.00	0.00	0.51	0.00	0.00
390.10165	390.10632	-11.97	C18H18N2O8	Dopa-Betaxanthin	0.00	0.00	0.00	0.00	0.00	1.44	0.00

546.10657	546.10683	-0.47	C18H26O19	Alpha-D-Galactopyranuronosyl-(1-4)-Alpha-D-Galactopyranuronosyl-(1-4)-D-Galacturonic Acid	0.00	30.23	46.76	121.23	0.00	0.00	0.00
274.19283	274.19328	-1.65	C18H26O2	Falcarindiol8-Methylether	0.00	0.00	27.16	5.67	0.00	0.00	0.00
278.22209	278.22458	-8.93	C18H30O2	A-Linolenic Acid	176.58	86.20	123.38	105.17	4.53	25.47	615.15
504.16643	504.16903	-5.17	C18H32O16	Raffinose	0.00	0.00	0.00	8.94	1.10	0.00	0.00
282.25126	282.25588	-16.36	C18H34O2	Oleic Acid	54.35	0.00	0.00	0.00	0.00	5.73	195.77
380.25802	380.25658	3.76	C18H39NO5P	Sphinganine 1-Phosphate	0.00	0.00	0.00	0.00	0.00	0.00	5.32
434.04733	434.04853	-2.75	C19H14O12	Ellagic Acidarabinoside	0.00	0.00	0.00	0.00	0.00	2.06	0.00
422.09135	422.08491	15.26	C19H18O11	Mangiferin	0.00	0.00	0.00	0.00	1.81	2.13	0.00
342.10887	342.11034	-4.30	C19H18O6	Tetramethylscutellarein	0.00	10.21	3.92	4.99	5.58	3.47	0.00
344.12735	344.12599	3.95	C19H20O6	Dehydrocyanaropicrin	0.00	0.00	0.00	0.00	0.34	0.00	0.00
360.11903	360.12090	-5.19	C19H20O7	Gibberellin A8-Catabolite	0.00	0.00	0.00	0.00	2.33	3.29	0.00
378.12954	378.13147	-5.09	C19H22O8	Oleuropein-Aglycone	47.17	81.61	5.02	0.00	72.85	129.33	13.78
354.11092	354.11034	1.63	C20H18O6	Sesamin	0.00	0.00	46.18	76.68	0.31	9.96	0.00
386.10095	386.10017	2.03	C20H18O8	8 5-Diferulic Acid	0.00	0.00	0.00	0.00	0.00	2.66	0.00
435.09451	435.09274	4.08	C20H19O11	Delphinidin-3-Arabinoside	0.00	0.00	107.76	160.34	0.00	4.18	0.00
403.09709	403.10291	-14.42	C20H19O9	Pelargonidin-3-Arabinoside	0.00	0.00	0.00	0.00	0.59	0.00	0.00
374.13643	374.13655	-0.32	C20H22O7	7-Hydroxymatairesinol	0.00	21.06	0.00	0.00	7.70	0.00	0.00
332.20132	332.19876	7.70	C20H28O4	Carnosic Acid	8.90	0.00	0.00	0.00	0.33	0.00	0.00
306.25327	306.25588	-8.54	C20H34O2	Akhdardiol	40.33	0.00	142.51	93.53	0.00	1.82	103.56
363.10947	363.11067	-3.31	C21H17NO5	Dihydrochelirubine	0.00	0.00	0.00	0.00	0.00	6.83	0.00
379.10797	379.10559	6.29	C21H17NO6	12-Hydroxydihydrochelirubine	0.00	31.85	0.00	0.00	0.00	0.00	0.00
431.09705	431.09782	-1.78	C21H19O10	Kaempferol3-O-Rhamnoside	0.00	0.00	21.94	26.48	0.00	0.00	0.00
464.09507	464.09548	-0.88	C21H20O12	Isoquercitrin	0.00	0.00	0.00	0.00	0.40	0.00	0.00
416.10473	416.11073	-14.42	C21H20O9	Daidzin	0.00	0.00	0.00	0.00	0.00	3.60	0.00
465.09844	465.10330	-10.44	C21H21O12	Delphinidin-3-Glucoside	0.00	0.00	0.00	0.00	0.00	2.39	0.00

402.13156	402.13147	0.24	C21H22O8	Nobiletin	0.00	24.79	4.23	5.83	1.24	8.28	56.73
435.12573	435.12912	-7.79	C21H23O10	Phlorizin	0.00	0.00	5.97	7.39	0.00	4.56	0.00
452.12349	452.13186	-18.52	C21H24O11	Aspalathin	4.86	0.00	0.00	0.00	0.00	0.00	4.67
388.14944	388.15220	-7.13	C21H24O7	Medioresinol	0.00	0.00	0.00	0.00	0.00	2.00	0.00
328.21022	328.20384	19.42	C21H28O3	16-O-Methylkahweol	0.00	0.00	5.59	0.00	0.00	0.00	0.00
499.15518	499.16245	-14.56	C21H29N3O9S	S-Sinapylglutathione	0.00	0.00	0.00	0.00	0.00	4.26	0.00
460.09268	460.09764	-10.79	C21H34Br2O	O,15-cyclo-14-bromo-14,15-dihydrorogiol-3,11-diol	0.00	0.00	0.00	0.00	0.00	0.00	5.79
492.08503	492.09039	-10.89	C22H20O13	Isorhamnetin3-O-Glucuronide	0.00	0.00	0.00	0.00	0.00	1.58	0.00
456.14938	456.15114	-3.86	C22H33BrO5	Isoparguerol	0.00	4.29	0.00	0.00	0.52	7.12	0.00
364.25714	364.26136	-11.58	C22H36O4	Dictyoepoxide	0.00	0.00	0.00	0.00	0.00	0.00	6.73
458.11852	458.12130	-6.05	C23H22O10	6-O-Acetyldaidzin	0.00	0.00	0.00	0.00	1.98	0.00	0.00
506.11294	506.10604	13.63	C23H22O13	Quercetin-3-(6-Acetylglucoside)	0.00	11.94	41.99	98.28	1.07	5.11	0.00
491.12594	491.11895	14.22	C23H23O12	Cyanidin3-O-(6-Acetyl-Glucoside)	0.00	11.72	38.10	90.41	0.00	4.17	0.00
476.12760	476.13186	-8.96	C23H24O11	Cirsimarin	0.00	18.70	0.00	0.00	2.84	0.00	0.00
528.09716	528.10345	-11.92	C23H25ClO12	Malvidin3-O-Galactoside	0.00	0.00	0.00	17.54	0.00	0.00	0.00
507.16406	507.16148	5.08	C23H27N2O11	2-Decarboxy-Betanin	0.00	0.00	0.00	0.00	0.00	1.35	0.00
378.27703	378.27701	0.05	C23H38O4	Persenonea	0.00	0.00	0.00	0.00	0.00	0.00	9.88
534.10098	534.10096	0.05	C24H22O14	Kaempferol3-O-(6-Malonyl-Glucoside)	13.68	619.05	795.20	1443.82	3.26	195.86	0.00
550.10073	550.09587	8.84	C24H22O15	Quercetin3-O-(6-Malonyl-Glucoside)	0.00	0.00	0.00	0.00	0.00	1.25	0.00
488.13896	488.13186	14.54	C24H24O11	6-O-Acetylglycitin	0.00	22.56	73.47	214.33	0.71	5.53	0.00
420.25128	420.25119	0.22	C24H36O6	Amijiol-7,10-diacetate	0.00	0.00	0.00	0.00	0.00	0.00	4.66
406.26661	406.27192	-13.09	C24H38O5	18,19-epoxyxenic-19-methoxy-18-hydroxy-4-acetoxy-6,9,13-triene	18.52	0.00	0.00	0.00	0.00	1.50	34.30
533.12937	533.12952	-0.26	C25H25O13	Pelargonidin3-O-(6-Succinyl-Glucoside)	0.00	0.00	0.00	0.00	0.00	1.24	0.00
394.21203	394.21441	-6.02	C25H30O4	Bixin	0.00	0.00	0.00	0.00	0.00	0.00	3.40

374.31214	374.31848	-16.94	C25H42O2	5-Nonadecenylresorcinol	4.70	0.00	0.00	0.00	0.33	0.00	0.00
580.26408	580.27311	-15.56	C26H44O14	3-Isobutanoyl-3,4-Di(3-Methylbutanoyl)Sucrose	9.17	0.00	0.00	0.00	0.00	0.00	0.00
594.16962	594.15847	18.77	C27H30O15	Kaempferol-3-O-Galactoside7-O-Rhamnoside	28.91	0.00	0.00	0.00	0.00	0.00	0.00
579.16347	579.17138	-13.66	C27H31O14	Pelargonidin3-O-Rutinoside	0.00	0.00	0.00	9.09	0.00	0.00	0.00
458.27504	458.26684	17.90	C27H38O6	Sargathunbergol A	4.57	56.95	10.40	8.07	19.49	57.31	22.48
384.33273	384.33922	-16.86	C27H44O	Desmosterol	36.36	0.00	0.00	0.00	0.00	2.65	5.66
447.33250	447.33486	-5.28	C27H45NO4	Esculeogeninb	0.00	0.00	29.04	5.08	0.00	0.00	0.00
594.29180	594.28876	5.11	C27H46O14	3-(2-Methylbutanoyl)-3,4-Di(3-Methylbutanoyl)Sucrose	0.00	0.00	0.00	0.00	0.00	0.00	4.62
580.31344	580.30949	6.80	C27H48O13	3-Decanoyl-4-(3-Methylbutanoyl)Sucrose	21.56	0.00	15.25	7.72	0.00	0.00	0.00
624.17500	624.16903	9.55	C28H32O16	Isorhamnetin3-O-Glucoside7-O-Rhamnoside	0.00	0.00	0.00	15.45	0.00	0.00	0.00
543.30340	543.29916	7.80	C28H47O8S	Castasterone 22-O-Sulfate	0.00	0.00	0.00	0.00	0.00	0.00	3.15
400.36386	400.37052	-16.62	C28H48O	Campesterol	2.96	0.00	0.00	0.00	0.00	2.93	0.00
450.37397	450.37091	6.79	C28H50O4	6-Hydroxyteasterone	28.93	0.00	0.00	0.00	0.59	11.68	7.63
564.12027	564.12678	-11.53	C29H24O12	Theaflavin	0.00	0.00	0.00	7.63	0.00	4.37	5.34
636.17081	636.16903	2.78	C29H32O16	Kaempferol3-O-(6-Acetyl-Galactoside)7-O-Rhamnoside	0.00	0.00	0.00	0.00	0.00	12.22	0.00
622.19552	622.18977	9.25	C29H34O15	Pectolinarin	0.00	0.00	0.00	0.00	0.63	0.00	0.00
579.15593	579.15025	9.80	C30H27O12	Pelargonidin-3-P-Coumaryl	0.00	0.00	11.23	27.44	0.00	0.00	0.00
454.34158	454.34470	-6.85	C30H46O3	Betulonic Acid	0.00	0.00	0.00	0.00	0.00	0.00	28.35
502.33508	502.32944	11.22	C30H46O6	Medicagenic Acid	0.00	0.00	0.00	0.00	0.45	0.00	11.53
456.36324	456.36035	6.34	C30H48O3	Betulinic Acid	0.00	0.00	0.00	0.00	4.86	0.00	11.25
488.35927	488.35017	18.62	C30H48O5	Asiatic Acid	0.00	0.00	0.00	0.00	0.00	5.96	117.34
428.40021	428.40182	-3.74	C30H52O	Cycloartanol	2.67	0.00	0.00	0.00	0.00	0.00	0.00
460.39220	460.39165	1.19	C30H52O3	Protopanaxadiol	15.99	0.00	0.00	0.00	0.00	2.22	3.83
508.37609	508.37639	-0.58	C30H52O6	(+)-Prelongilene	0.00	0.00	0.00	0.00	0.45	11.67	2.99

650.35622	650.35136	7.48	C31H54O14	3-Acetyl-3-Dodecanoyl-4-(3-Methylbutanoyl)Sucrose	0.00	0.00	0.00	0.00	10.43	0.00	0.00
726.20452	726.20073	5.22	C32H38O19	Pelargonidin-3-Sambubioside-5-Glucoside	0.00	8.89	53.27	155.38	0.00	0.00	0.00
741.22183	741.22420	-3.21	C33H41O19	Pelargonidin3-O-Glucosyl-Rutinoside	0.00	0.00	19.19	55.87	0.00	3.16	0.00
757.22184	757.21912	3.59	C33H41O20	Cyanidin3-O-Glucosyl-Rutinoside	0.00	0.00	0.00	12.03	0.51	0.00	0.00
582.20246	582.21175	-15.95	C34H30MgN4O4	Mg-Protoporphyrin	7.21	0.00	0.00	0.00	0.00	0.00	0.00
684.27405	684.27819	-6.05	C36H44O13	Mascarosideiii	0.00	232.52	13.83	36.49	6.95	6.49	0.00
580.44823	580.44916	-1.60	C38H60O4	Cafestololeate	0.00	0.00	0.00	0.00	0.00	0.00	3.79
618.39215	618.39204	0.19	C39H54O6	Alphitolic Acid (3-O-Cis-P-Coumaroyl-)	0.00	0.00	13.67	0.00	1.08	0.00	0.00
592.45075	592.44916	2.69	C39H60O4	Stigmastanol Ferulate	15.32	3.12	62.48	56.20	0.00	4.33	17.02
564.39207	564.39673	-8.26	C40H52O2	Canthaxanthin	17.39	0.00	21.27	11.66	0.00	4.77	0.00
552.43253	552.43312	-1.07	C40H56O	Cryptoxanthin-Alpha	8.84	0.00	0.00	0.00	0.00	1.95	14.53
600.42595	600.41786	13.48	C40H56O4	Neoxanthin	21.85	0.00	8.12	0.00	0.00	6.85	17.03
708.28477	708.29345	-12.25	C42H44O10	Paradisin B	0.00	0.00	38.35	177.73	0.00	0.00	0.00
800.49302	800.49221	1.01	C42H72O14	Ginsenosiderf	0.00	0.00	0.00	0.00	0.88	0.00	0.00
936.43119	936.42022	11.71	C43H68O22	Rebaudioside F	0.00	4.43	47.28	35.85	0.00	0.00	0.00
746.48807	746.49690	-11.83	C43H70O10	Monogalactosyldiacylglycerol	0.00	6.53	0.00	0.00	0.00	0.00	0.00
979.27751	979.27194	5.69	C44H51O25	Cyanidin3-(Sinapoyl)-Diglucoside-5-Glucoside	9.21	39.04	0.00	0.00	0.69	2.40	0.00
898.19177	898.19564	-4.31	C45H38O20	Prodelphinidintrimergc-Gc-C	19.06	16.62	20.95	25.65	0.00	0.00	0.00
852.50918	852.51093	-2.06	C45H74NO14	Alpha-Chaconine	0.00	27.62	438.44	325.18	3.79	0.00	20.89
118.02729	118.02661	5.80	C4H6O4	Succinic Acid	0.00	0.00	0.00	0.00	0.00	0.00	2.96
134.02119	134.02152	-2.46	C4H6O5	Malic Acid	0.00	4.65	0.00	0.00	22.37	3.22	0.00
1141.34512	1141.32477	17.83	C50H61O30	Cyanidin3-(Sinapoyl)-Triglucoside-5-Glucoside	0.00	12.72	0.00	0.00	0.00	0.00	0.00
1317.36003	1317.37211	-9.17	C60H69O33	Cyanidin3-(Feruloyl)(Sinapoyl)-Triglucoside-5-Glucoside	0.00	70.25	0.00	0.00	0.00	0.00	0.00

1343.37067	1343.35137	14.37	C61H67O34	Cyanidin 3-O-(2-O-(2-O-(Sinapoyl) Xylosyl) 6-O-(P-O-(Glucosyl)-P-Coumaroyl) Glucoside) 5-O-(6-O-(Malonyl) Glucoside	0.00	0.00	0.00	5.42	0.00	0.00	0.00
180.06178	180.06339	-8.92	C6H12O6	D-Fructose	0.00	0.00	0.00	0.00	7.67	3.64	101.97
196.05746	196.05830	-4.31	C6H12O7	Gluconic Acid	0.00	4.38	0.00	0.00	0.00	0.00	0.00
174.01578	174.01644	-3.79	C6H6O6	L-Dehydro-Ascorbate	0.00	0.00	0.00	0.00	0.00	1.48	0.00
208.02101	208.02192	-4.35	C6H8O8	D-Glucarate	0.00	0.00	0.00	0.00	0.00	0.00	3.33
176.06766	176.06847	-4.65	C7H12O5	2-Isopropylmalic Acid	0.00	32.42	0.00	0.00	0.00	0.00	0.00
212.08947	212.08960	-0.64	C7H16O7	Perseitol	0.00	0.00	5.39	8.08	0.58	0.00	91.40
231.93732	231.93712	0.88	C7H5BrO4	3-bromo-4,5-dihydroxybenzaldehyde	0.00	0.00	0.00	0.00	0.34	0.00	6.92
152.03439	152.03477	-2.47	C7H6NO3	5-Hydroxyanthranilate	0.00	0.00	0.00	0.00	0.00	0.00	3.60
122.03617	122.03678	-4.98	C7H6O2	Benzoic Acid	6.01	0.00	8.69	0.00	16.58	11.95	3.43
203.05971	203.06161	-9.36	C8H13NO3S	S-Allylmercapturic Acid	0.00	0.00	7.65	5.23	1.51	0.00	0.00
141.11586	141.11536	3.49	C8H15NO	Physoperuvine	0.00	0.00	8.00	0.00	0.00	0.00	0.00
323.85872	323.86328	-14.09	C8H6Br2O4	2-(2,3 -dibromo- 4,5-dihydrox yphenyl)acetic acid	0.00	0.00	0.00	0.00	0.32	0.00	0.00
244.07169	244.06954	8.82	C9H12N2O6	Uridine	0.00	3.00	13.87	23.39	0.32	3.97	6.64
188.10601	188.10486	6.10	C9H16O4	Azelaic Acid	9.21	0.00	0.00	0.00	0.00	0.00	9.69

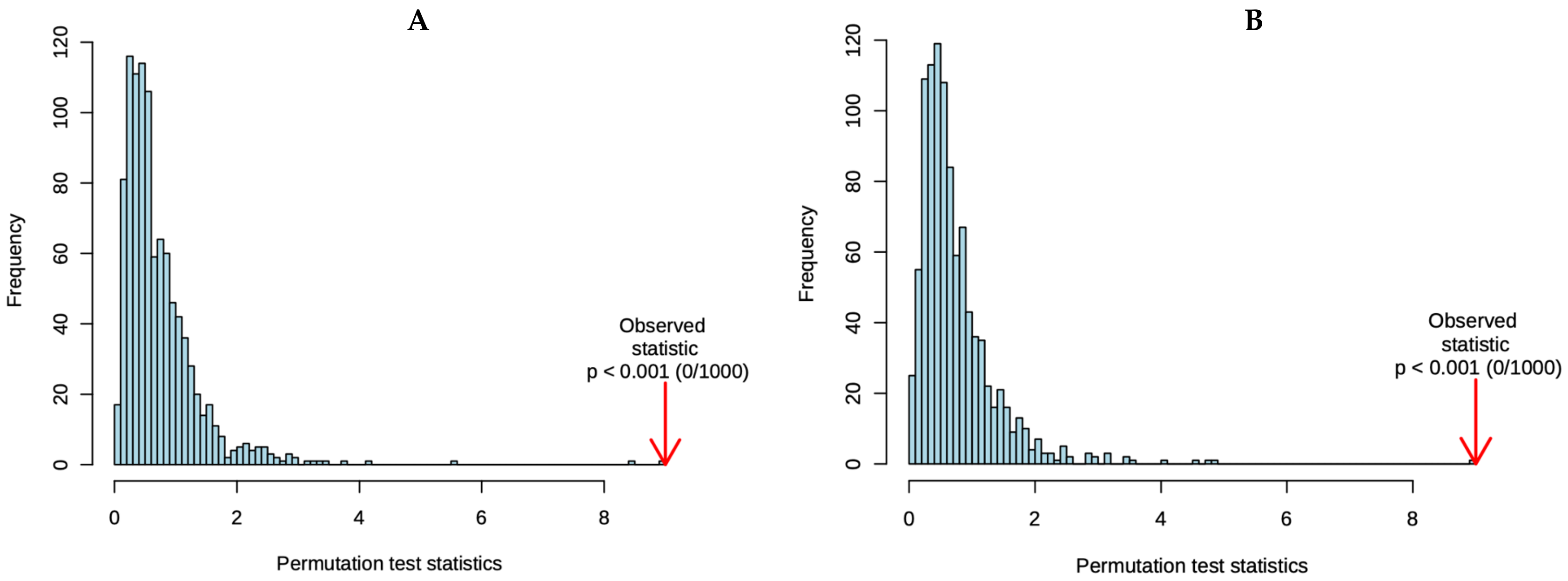

Figure S1: PLS-DA model validation in A) positive and B) negative ESI modes datasets by permutation tests based on separation distance. The p value based on permutation is $p < 0.001$ (0/1000).
